# Association between *Eubacterium saphenum* and periodontitis: A systematic review

**DOI:** 10.1371/journal.pone.0357110

**Published:** 2026-09-02

**Authors:** Sarah Al-Rihaymee, Hayder Raad Abdulbaqi, Ali A. Abdulkareem, Wan Himratul Aznita Wan Harun, Nor Adinar Baharuddin

**Affiliations:** 1 Department of Restorative Dentistry, Faculty of Dentistry, Universiti Malaya, Kuala Lumpur, Malaysia; 2 Department of Periodontics, College of Dentistry, University of Baghdad, Baghdad, Iraq; 3 Department of Oral and Craniofacial Sciences, Faculty of Dentistry, Universiti Malaya, Kuala Lumpur, Malaysia; University of Granada: Universidad de Granada, SPAIN

## Abstract

**Objective:**

Although established periodontal pathobionts are frequently detected, their presence alone does not fully explain variations in disease severity, highlighting the need to identify additional microbial contributors. This systematic review evaluates the available evidence on the association between *Eubacterium saphenum* and the severity of periodontitis.

**Methods:**

The review was conducted in accordance with the PRISMA 2020 framework and registered in PROSPERO (CRD420251273182). Observational studies involving systemically healthy adults were included that compared individuals with periodontitis and periodontal health, reporting detection and/or relative abundance of *E. saphenum* in saliva and/or subgingival biofilm samples. Study quality was appraised using the AXIS tool, and certainty of evidence was assessed using the GRADE framework.

**Results:**

Eight observational studies (304 healthy controls vs. 510 periodontitis cases) from Asia, Europe, and the Americas met the inclusion criteria. Across multiple detection platforms, *E. saphenum* showed a qualitatively similar direction of association, being more frequently detected and/or present at higher relative abundance in periodontitis than in periodontal health controls. Among studies applying the 2018 periodontal classification system and reporting stage-specific analyses, higher detection or enrichment of *E. saphenum* was generally observed in advanced disease (stages III-IV). The certainty of evidence was rated as low owing to the observational and predominantly cross-sectional design of the evidence, methodological heterogeneity, and lack of temporality.

**Conclusions:**

Low-certainty evidence suggests a positive directional association between *E. saphenum* and periodontitis, particularly in advanced Stage III–IV disease. However, longitudinal, interventional, and mechanistic studies are required to clarify temporality and determine whether *E. saphenum* contributes functionally to periodontal dysbiosis or primarily represents a marker of established disease.

## 1. Introduction

Periodontitis is initiated by the polymicrobial biofilm, but disease progression reflects a dysregulated host response largely determined by genetic and environmental factors [1–3]. Although established periodontal pathobionts, including *Porphyromonas gingivalis*, *Tannerella forsythia*, *Treponema denticola*, *Aggregatibacter actinomycetemcomitans*, *Fusobacterium nucleatum*, and *Filifactor alocis*, have been widely implicated in periodontitis, their detection alone does not fully explain inter-individual variation in disease severity or progression, supporting a community-level dysbiosis model of disease [4–9]. Contemporary models therefore emphasise polymicrobial synergy and dysbiosis, in which shifts in microbial community structure and function, together with host inflammatory responses, drive periodontal tissue destruction rather than the activity of a single causative species [10,11]. This paradigm highlights the need to investigate non-classical microbial taxa that may act as markers of disease severity or as members of dysbiotic microbial consortia within the subgingival ecosystem. Within this context, emerging non-classical anaerobic taxa such as *E. saphenum* deserve focused evaluation because they may provide additional insight into dysbiotic pocket ecology and disease-severity patterns beyond the classical periodontal pathogen complexes.

*E. saphenum* is a Gram‑positive obligate anaerobe, originally isolated from human periodontal pockets [12]. Its relevance to periodontitis is biologically plausible because it is suited to the low-redox, anaerobic environment characteristic of deep periodontal pockets and has been associated with asaccharolytic and proteolytic oral microbial communities [12,13]. Although *E. saphenum* is not traditionally included among established periodontal pathogens, it has been increasingly detected through molecular diagnostic approaches, including PCR-based assays and high-throughput sequencing platforms [14–16]. More recent studies have reported *E. saphenum* enrichment in periodontitis, particularly in advanced disease [17,18]. Several clinically and biologically relevant considerations support the appraisal of *E. saphenum* as an emerging non-classical periodontal taxon. First, it occupies an ecological niche directly relevant to advanced periodontal dysbiosis. Second, it has been repeatedly detected in periodontitis despite not being part of the classical periodontal pathogen groups. Third, the available evidence remains fragmented across different populations, sample types, periodontal diagnostic criteria, and microbial detection platforms. Therefore, the key gap is whether the repeated detection of *E. saphenum* reflects a reproducible severity-associated component of periodontal dysbiosis, particularly in advanced periodontitis, and how this taxon should be interpreted within the broader polymicrobial dysbiosis framework. Given the observational nature of the available evidence, findings in this review were interpreted as associations rather than evidence of causality. Accordingly, this review interprets *E. saphenum* within an ecological dysbiosis framework, distinguishing evidence for association from evidence for temporality, pathogenicity, or causal contribution. We hypothesised that *E. saphenum* would be more frequently detected or enriched in periodontitis than in periodontal health and that its detection or abundance would be higher in advanced disease, supporting its potential role as a marker of dysbiotic progression rather than as a confirmed primary pathogen.

Focused Question

Is *E. saphenum* associated with periodontitis compared with periodontal health, and does its abundance increase with disease severity?

## 2. Methods

The present systematic review was conducted and reported in accordance with the recommendations of the PRISMA 2020 (Preferred Reporting Items for Systematic Reviews and Meta-Analyses) statement [19]. The study was prospectively registered with PROSPERO (ID: CRD420251273182).

PECO: Population: systemically healthy adult participants; Exposure: individuals diagnosed with periodontitis (based on recognised clinical classification systems, including the 1999 AAP or 2018 EFP/AAP frameworks); Comparator: individuals with periodontal health; Outcome: detection, prevalence or relative abundance of *E. saphenum* in subgingival biofilm samples and/or saliva samples. Eligible study designs included observational studies (cross-sectional and case–control studies) reporting original clinical data.

### 2.1. Eligibility criteria

Only the studies that met the following criteria were considered: availability of full text online; *in vivo* original articles; the laboratory confirmation of the presence of *E. saphenum*; systemically healthy participants; comparison of healthy controls versus periodontitis; or presenting an unambiguous assessment of the association between *E. saphenum* and periodontitis. Studies were excluded if they involved non-human participants, lacked a comparison between periodontitis and healthy groups, or reported outcomes irrelevant to periodontitis or *E. saphenum*; had unclear definitions of periodontitis; or were case reports/series, review articles, or *in vitro* studies.

### 2.2. Search strategy

**Fig 1:**
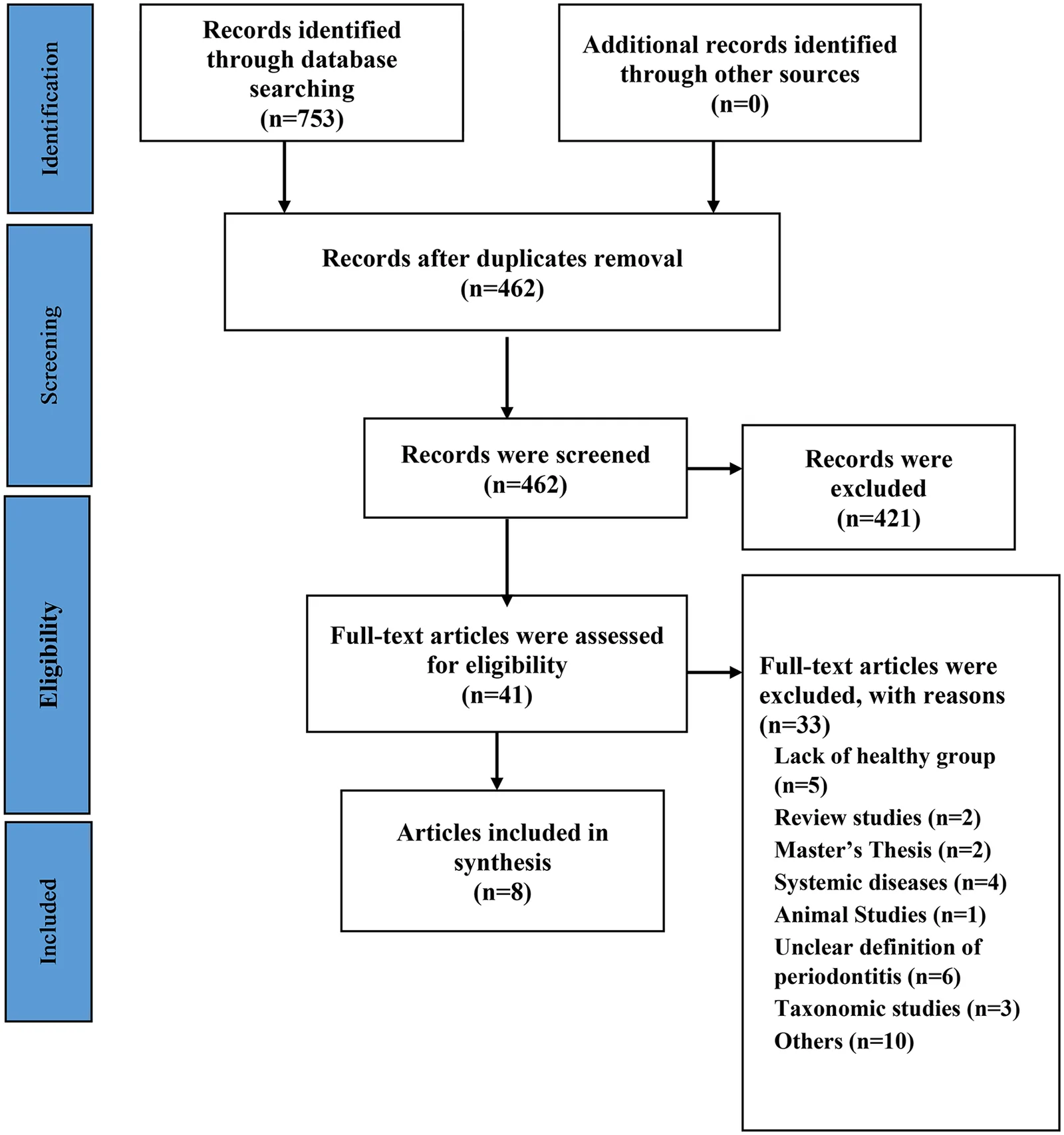
PRISMA flow diagram of the study-selection process. n denotes the number of records or studies, as applicable.

A comprehensive search of PubMed/MEDLINE, Scopus, Web of Science Core Collection, the Cochrane Library, and Google Scholar was conducted between June and September 2025, with no restrictions on publication date. For the Google Scholar search results, the first 200 references, representing the first 20 pages, were screened [20]. The keywords used in the search were: (*Eubacterium saphenum* OR *E. saphenum* OR *E*. *saphenus*) AND (periodontitis OR periodontal disease). The records of all sources were exported in RIS/BibTeX format and merged in Zotero. Duplicates (i.e., those identified by the search strategy across multiple databases) were removed in Zotero and Rayyan using a hierarchical protocol as follows: 1) exact DOI or PMID/PMCID match; 2) normalise title, first author, and publication year; and 3) screen the records based on titles and abstracts. After removing duplicates, all ineligible articles were excluded, with the reasons for exclusion recorded. A total of eight eligible articles were included in the final review, as shown in the PRISMA flow chart (Fig 1).

### 2.3. Data extraction

The data from each study were extracted and synthesised by two researchers (S.A. and H.R.A.) in an independent and standardised manner. Additionally, the studies were screened by an independent reviewer (A.A.A). For each study included, the following data were collected: (1) author(s) and year of publication, (2) location of the study, (3) study design, (4) periodontal diagnosis as defined in each article, (5) population (sample size), (6) type of sample (subgingival dental biofilm and/or saliva), (7) detection methods, (8) association of *E. saphenum* with periodontitis severity, (9) quantitative/statistical finding, and (10) main findings.

### 2.4. Quality assessment, risk of bias, and Certainty of Evidence of the included articles

The quality assessment score and the transparency of the reporting of all eligible studies in this review were critically evaluated utilising the AXIS tool [21]. The AXIS tool was selected because the included studies were observational and predominantly cross-sectional in design, involving comparisons of microbial detection or relative abundance between periodontal health and periodontitis groups. Although the Joanna Briggs Institute checklist is also appropriate for analytical cross-sectional studies, AXIS was chosen because it provides a broader 20-item framework for evaluating both methodological quality and reporting transparency, including sample-size justification, sampling frame, representativeness, non-response, limitations, ethical approval, and potential conflicts of interest. This approach is consistent with methodological guidance recommending that appraisal tools should be selected according to the design and methodological characteristics of the included studies [22].

The AXIS tool, designed for observational studies, comprises twenty questions with a possible response of yes, no, or don't know for each question (for calculation purposes, yes = 1, no/don't know = 0). A quality score out of 20 was then generated based on the sum of these responses. The following guidelines were used: scores indicating low quality = 1–7; medium quality = 8–14; high quality = 15–20. A quantitative meta-analysis was not performed because the included studies showed substantial clinical and methodological heterogeneity in sample matrix, microbial detection platform, periodontal case definition, disease-severity classification, and microbial outcome reporting. Instead, a structured qualitative synthesis was performed. Studies were compared according to five predefined domains: biological sample type, detection method, periodontal diagnostic framework, microbial outcome metric, and availability of severity-specific analysis. To reduce subjectivity in the qualitative synthesis, these domains were extracted systematically for each included study. The biological sample type was classified as saliva or subgingival biofilm. The detection platform was classified as qPCR, 16S rRNA sequencing, HOMIM microarray, or chequerboard DNA-DNA hybridisation. Periodontal diagnosis was classified according to whether studies used the 2018 EFP/AAP classification, the 1999 AAP framework, or study-specific clinical thresholds. Microbial outcomes were classified as detection, prevalence, relative abundance, absolute quantification, or correlation with clinical periodontal parameters. Severity-specific analysis was recorded where studies compared early/mild disease with advanced periodontitis.

The certainty of evidence for the association between *E. saphenum* and periodontitis was assessed using the GRADE approach (Grading of Recommendations, Assessment, Development and Evaluation) at the outcome level. The body of evidence was judged to start at low certainty because the included studies were observational, and it was then assessed across the standard GRADE domains: risk of bias, inconsistency, indirectness, imprecision, and publication bias. Potential upgrading factors, including large estimates and severity-related patterns, were considered descriptively. However, because cross-study agreement could only be assessed qualitatively across heterogeneous methodologies, consistency was not used as a formal upgrading criterion. Final certainty ratings (very low/low/moderate/high) were assigned to the body of evidence for the outcome [23,24].

## 3. Results

### 3.1. Included studies

Following PRISMA guidelines, database searches identified 753 records, with no additional sources retrieved. After deduplication, a total of 462 unique records remained, which were then screened by title and abstract, resulting in the exclusion of 421 records. The remaining forty-one full-text articles were then assessed for eligibility. Thirty-three articles were excluded for the following predefined reasons: absence of a healthy control group (n = 5), review articles (n = 2), master's theses (n = 2), systemic diseases (n = 4), animal studies (n = 1), taxonomic studies (n = 3), (n = 6) for studies with an unclear definition of periodontitis, and (n = 10) for other reasons, including studies of (pregnant women, elderly populations, laboratory-only investigations, root-canal research, and peri-implantitis). Ultimately, eight studies that met the inclusion criteria were incorporated into the qualitative synthesis, as shown in (Fig 1).

### 3.2. Study designs, periodontal conditions/samples evaluated, and examiner calibration

The included studies were conducted in Asia (China and South Korea), Europe (Spain and Sweden), and the Americas (USA, Brazil, and Colombia). The study arms consisted of periodontal health controls and patients with periodontitis, with a total of 304 and 510 participants, respectively. Clinical heterogeneity was evident across the included studies. Although all studies compared periodontal health with periodontitis, they did not use identical diagnostic frameworks. Four studies used the 2018 EFP/AAP classification, two studies used the 1999 AAP framework, and two studies used study-specific clinical definitions based on combinations of probing pocket depth, clinical attachment loss, bleeding on probing, and radiographic bone loss. This variation limits direct comparability between studies because “periodontitis” did not represent an identical clinical construct across all included investigations. Saliva or subgingival biofilm samples were used to assess *E. saphenum* detection, abundance, or microbial profiles. Two studies collected saliva samples from 80 periodontal health participants and 74 participants with periodontitis [25,26]. A total of 224 and 436 subgingival biofilm samples were collected from participants with periodontal health and periodontitis, respectively [16–18,27–29], Table 1. These subgingival biofilm samples were processed either individually in one study [27] or pooled in four studies [16–18,28].

**Table 1:** Summary of the publications included for analysis of the association between *E. saphenum* and periodontitis.

Study/Year of publication	Country	Healthy (n)	Periodontitis (n)	Type of sample	Method to identify *E. saphenum*	Periodontal diagnosis (as defined in the paper)	Association of *E. saphenum* with periodontitis severity	Quantitative/statistical finding	Main findings
[26]	Sweden	47	46	Saliva	16S rRNA gene sequencing	BOP > 30%, sites with PPD ≥ 6 mm, CAL ≥ 5 mm with radiographic examination	Not assessed	RF accuracy 79.5%.	*E. saphenum* was higher in periodontitis than in periodontal health.
[25]	South Korea	33	28	saliva	16S rRNA amplicon sequencing	**2018 periodontal** classification [31]	Not assessed	Increased in periodontitis vs healthy; Bonferroni p < 0.05.	*E. saphenum* was higher in periodontitis than in periodontal health.
[17]	Spain & Colombia	20	40	subgingival biofilm	16S rRNA amplicon sequencing	2018 **periodontal** classification [1]	Reported	Health vs Stage I–II: −1.495, p = 0.002; Health vs Stage III–IV: −2.158, p = 0.001; Stage I–II vs III–IV: NS	*E. saphenum* was higher in periodontitis than in periodontal health.
[27]	Brazil	81	134	subgingival biofilm	Checkerboard DNA–DNA hybridisation	AAP classification. Chronic periodontitis (CP): > 10% teeth with PD and/or CAL ≥ 5 mm with BOP. Generalised aggressive (AgP): ≥ 30% teeth with PD/CAL ≥ 5 mm with BOP [30,33]	Not assessed	Mean counts correlated with PD: rho = 0.151, p = 0.022; other correlations NS.	A significant correlation with periodontal clinical parameters.
[18]	Spain & Colombia	60	120	subgingival biofilm (paper points)	Targeted qPCR	2018 **periodontal** classification [1,31,32]	Reported	Stage III–IV vs health: OR = 4.85, 95% CI 1.99–11.7.	*E. saphenum* was higher in periodontitis than in periodontal health.
[16]	Spain & Colombia	18	37	Subgingival biofilm (paper points)	qPCR assay	2018 **periodontal** classification [1,31,32]	Reported	qPCR detection: 43/55 samples (78.2%)	*E. saphenum* was higher in periodontitis than in periodontal health.
[29]	USA	30	90	Subgingival biofilm (paper points)	HOMIM microarray	periodontitis: ≥ 8 sites with radiographic bone loss, ≥ 8 sites with PD > 4 mm, and >30% of sites CAL > 3 mm [34]	Not assessed	Correlation with microbial community: r = 0.567, p ≤ 0.05; community vs PD r = 0.181, p = 0.025; BOP r = 0.215, p = 0.008	*E. saphenum* significantly was correlated with PPD and BOP.
[28]	China	15	15	Subgingival biofilm (paper points)	HOMIM microarray	AAP 1999 aggressive periodontitis framework ≥ 4 sites with PPD ≥ 5 mm, CAL ≥ 2 mm, and BOP; ≥ 20 teeth [35]	Not assessed	Higher abundance in aggressive periodontitis: W = 3.97, p < 0.001; PPD coefficient = 0.496, p = 0.047	*E. saphenum* was higher in periodontitis than in periodontal health.

BOP: bleeding on probing; PD/PPD: probing pocket depth; CAL: clinical attachment loss; qPCR: quantitative PCR; HOMIM microarray: Human Oral Microbe Identification Microarray. OR = odds ratio; CI = confidence interval; RF = random forest. NS = not statistically significant; rho = Spearman correlation strength. The OR from Lafaurie et al., 2023 [18] is the unadjusted estimate.

The definition of periodontitis cases varied across the included studies. Two studies [26,29] defined periodontitis using probing pocket depth (PPD) thresholds (PPD ≥ 4–6 mm) and bleeding on probing (BOP), alongside CAL and radiographic bone loss [30]. Two other studies used the 1999 AAP framework [27,28]. The 2018 EFP/AAP staging system [1,31,32] was applied by four of the studies [16–18,25], establishing comparisons according to periodontitis severity (stages I-II vs. III-IV), Table 1.

Among the included studies, examiner calibration was reported in only five studies [16–18,27,28]. Reducing measurement errors in recording PPD and CAL is necessary to define cases and sampling sites.

### 3.3. Method of detection

The included studies showed substantial heterogeneity in the detection methods of *E. saphenum*. Several investigations utilised 16S rRNA gene sequencing to characterise bacterial communities [17,25,26]. In contrast, Vieira Colombo et al. (2016) applied checkerboard DNA–DNA hybridisation targeting 39 bacterial taxa, including *E. saphenum* [27]. Other studies focused on targeted quantitative PCR (qPCR) for specific pathogen detection, as reported by Lafaurie et al. (2023) and Castillo et al. (2023), both of which collected subgingival biofilm samples by paper points [16,18]. Additionally, Cui et al. (2019) implemented a Human Oral Microbe Identification Microarray (HOMIM) based on 16S species-specific probes to evaluate bacterial composition in samples from generalised aggressive periodontitis cases [28]. Similarly, Marchesan et al. (2015) used the HOMIM microarray to profile the periodontal microbial community [29]. Overall, variable methods were utilised in these studies for detecting *E. saphenum,* as shown in Table 1. These platform differences may introduce detection bias because targeted methods, such as qPCR, HOMIM and chequerboard DNA–DNA hybridisation, depend on predefined primers or probes, whereas 16S rRNA sequencing is affected by primer choice, amplified region, sequencing depth, database selection and bioinformatic pipeline [18,25,27–29]. Consequently, qPCR copy number, HOMIM/chequerboard signal intensity and sequence-derived relative abundance should not be interpreted as directly equivalent outcomes.

### 3.4. Microbial data

A structured qualitative synthesis was undertaken to summarise the direction and type of evidence across the included studies. At the study level, all eight included studies contributed findings in a positive qualitative direction for *E. saphenum* in relation to periodontitis, advanced disease, or periodontal disease-associated microbial profiles. However, the studies supported this pattern in different ways. Some studies reported increased detection, abundance, or enrichment in periodontitis or advanced disease, whereas others reported correlations with periodontal clinical parameters or microbial-community associations. Severity-specific evidence was available in three studies, all of which supported higher detection or enrichment of *E. saphenum* in advanced periodontitis. Some studies investigated subgingival biofilms and reported a higher prevalence of *E. saphenum* in diseased periodontal pockets than in periodontal health sites [16–18,28]. Additionally, two studies linked the abundance of *E. saphenum* with clinical parameters, indicating a positive correlation of the bacterium with increasing BOP and PPD [27,29]. In contrast, two studies observed elevated *E. saphenum* loads in the saliva of periodontitis patients [25,26]. Studies using the 2018 staging framework found a repeated association of *E. saphenum* with severe periodontitis (stages III-IV) [16–18,25]. Table 1 summarises each study’s sample size, detection method, diagnostic criteria, severity association, available quantitative/statistical indicators, and main findings.

### 3.5. Quality of studies and risk of bias

The quality assessment and risk of bias revealed that all included studies demonstrated varying levels of methodological quality and reporting transparency, as assessed by the AXIS evaluation. The 20-item AXIS checklist was used to assess methodological quality and reporting transparency, Table 2. The overall scores ranged from medium to high quality, reflecting differences in study design clarity, sample justification, and data presentation. According to the predefined criteria, four studies were of high quality, and the remaining four studies were rated as medium quality. Common limitations included the absence of sample-size justifications and uncertainties surrounding sampling frames. Aims, measurement methods, and the reporting of basic data were generally reliable, Table 3.

**Table 2:** Quality assessment of included studies using the AXIS tool checklist.

	AXIS Item	Lee 2025	Marchesan 2015	Castillo 2023	Cui 2019	Lafaurie 2023	Lundmark 2019	Vieira 2016	Lafaurie 2022
1.	Were the aims/objectives of the study clear?	Yes	Yes	Yes	Yes	Yes	Yes	Yes	Yes
2.	Was the study design appropriate for the stated aim(s)?	Yes	Yes	Yes	Yes	Yes	Yes	Yes	Yes
3.	Was the sample size justified?	Yes	DK*	DK	DK	Yes	Yes	DK	DK
4.	Was the target population clearly defined?	Yes	Yes	Yes	Yes	Yes	Yes	Yes	Yes
5.	Was the sample frame taken from an appropriate population base so that it closely represented the target population under investigation?	No	DK	DK	DK	DK	DK	DK	Yes
6.	Was the selection process likely to select participants that were representative of the target population under investigation?	DK	DK	DK	DK	DK	DK	DK	DK
7.	Were measures undertaken to address and categorize non-responders?	DK	DK	DK	DK	DK	DK	DK	DK
8.	Were the outcome variables measured appropriate to the aims of the study?	Yes	Yes	Yes	Yes	Yes	Yes	Yes	Yes
9.	Were the outcome variables measured correctly using measures that had been trialled, piloted, or published previously?	Yes	Yes	Yes	Yes	Yes	Yes	Yes	Yes
10.	Is it clear what was used to determine statistical significance and/or precision estimates? (e.g., p values, CIs)	Yes	Yes	Yes	Yes	Yes	Yes	Yes	Yes
11.	Were the methods (including statistical methods) sufficiently described to enable them to be repeated?	Yes	Yes	Yes	Yes	Yes	Yes	Yes	Yes
12.	Were the basic data described?	Yes	Yes	Yes	Yes	Yes	Yes	Yes	Yes
13.	Does the response rate raise concerns about nonresponse bias?	DK	DK	DK	DK	DK	DK	DK	DK
14.	If appropriate, was information about non-responders described?	DK	DK	DK	DK	DK	DK	DK	DK
15.	Were the results internally consistent?	Yes	Yes	Yes	Yes	Yes	Yes	Yes	Yes
16.	Were the results for the analyses described in the methods presented?	Yes	Yes	Yes	Yes	Yes	Yes	Yes	Yes
17.	Were the authors’ discussions and conclusions justified by the results?	Yes	Yes	Yes	Yes	Yes	Yes	Yes	Yes
18.	Were the limitations of the study discussed?	Yes	Yes	Yes	Yes	Yes	Yes	Yes	Yes
19.	Were there any funding sources or conflicts of interest that may affect the authors’ interpretation of the results?	Yes	Yes	Yes	DK	Yes	Yes	Yes	Yes
20.	Was ethical approval or consent of participants attained?	Yes	Yes	Yes	Yes	Yes	Yes	Yes	Yes

*DK = Don’t Know.

**Table 3:** AXIS scoring results.

Study	Total Score (0–20)	Quality Category
[16]	14	Medium
[28]	13	Medium
[17]	15	High
[18]	15	High
[25]	15	High
[26]	15	High
[29]	14	Medium
[27]	14	Medium

Using the GRADE framework to rate the certainty of evidence, the body of evidence started at low certainty and was then assessed across domains [23]. First, the risk of bias raised some concerns because four studies were rated as medium quality and four as high quality, with common limitations in sample-size justification and sampling frames. Second, inconsistency was judged not serious only at the level of qualitative direction of association; this judgement means that most studies pointed toward increased detection, enrichment, or clinical correlation of *E. saphenum*, not that effect sizes were homogeneous or directly comparable. Quantitative inconsistency could not be formally assessed because studies used heterogeneous sample types, detection platforms, case definitions, and outcome metrics. Third, indirectness was not serious because the studies aligned with the PECO criteria. Fourth, imprecision raised some concerns due to the modest individual study sample sizes and the absence of quantitative pooling. Finally, publication bias cannot be excluded. Potential upgrading factors were considered, including severity-related enrichment and the unadjusted odds ratio (OR = 4.85, 95% CI 1.99–11.7) / bivariate estimate reported by Lafaurie et al. [18]. However, no formal upgrade was applied because this estimate was unadjusted and derived from a single observational study; severity-specific evidence was limited; the evidence remained methodologically heterogeneous; cross-study consistency was qualitative rather than quantitative; and no included study established temporality. Therefore, the overall certainty of evidence was conservatively rated as low for the association between *E. saphenum* and periodontitis [24], as illustrated in Table 4.

**Table 4:** The overall certainty of evidence (GRADE) for the association between E. saphenum and periodontitis.

Domain	Decision	Rationale
**Outcome and Direction**	*E. saphenum* increased in periodontitis vs health, with some studies showing increased levels in Stage III–IV disease.	Overall positive directional pattern across heterogeneous saliva and subgingival-biofilm studies.
**Risk of bias**	Some concerns.	Several studies report calibration: AXIS quality ranged from medium to high across included studies.
**Inconsistency**	Not serious for qualitative direction only; quantitative inconsistency could not be assessed.	qualitative directional agreement
**Indirectness**	Not serious	Included studies matched the PECO question.
**Imprecision**	Some concerns	Modest sample sizes
**Publication bias**	Cannot be excluded	Diversity in methods and regions
**Upgrading factors**	Considered, but insufficient for upgrading	A moderate-to-large unadjusted association estimate (OR = 4.85) * was considered. However, no formal upgrade was applied, as the estimate was derived from a single observational study, while the overall body of evidence remained methodologically heterogeneous and unable to establish temporality.
**Overall certainty**	Low	Observational evidence started at low certainty and remained low because supportive findings were insufficient for formal upgrading. The evidence supports a low-certainty observational association, but not temporality, causality, or independent pathogenic contribution.

* Unadjusted Odds ratio / bivariate estimate reported by Lafaurie et al. 2023 [18] for the association between the presence of *E. saphenum* and Stage III–IV periodontitis compared with health, 95% CI 1.99–11.7. It was interpreted as supportive of the direction and magnitude of association.

## 4. Discussion

Rather than representing a standalone pathogen, *E. saphenum* is best conceptualised as a severity-associated microbial signal in polymicrobial periodontal dysbiosis. Its increased detection or abundance may reflect ecological shifts associated with established disease rather than an independent pathogenic role. This systematic review synthesised available observational evidence examining whether *E. saphenum* is enriched in periodontitis and whether its detection or abundance varies by disease severity. Across the included studies, *E. saphenum* was generally more frequently detected or enriched in advanced stages of periodontitis, supporting its interpretation as a low-certainty marker of dysbiotic pocket ecology. However, whether it contributes functionally within polymicrobial consortia remains unresolved.

These observations are consistent with ecological succession in the subgingival environment as disease progresses, rather than evidence that a single organism independently drives tissue destruction. As periodontal pockets deepen and oxygen tension declines, conditions increasingly favour obligate anaerobes and proteolytic species that utilise host-derived peptides. Within this shifting niche, *E. saphenum* may thrive alongside other dysbiosis-associated taxa, co-occurring within microbial communities that sustain inflammation and tissue breakdown. Members of the genus *Eubacterium* are recognised for their role in amino acid fermentation and short-chain fatty acid production, including metabolic activities that may be relevant to local inflammatory responses and also support biofilm maturation. Although the specific virulence mechanisms of *E. saphenum* remain unclear, its recurrent detection alongside established late colonisers suggests that it may function as part of a cooperative anaerobic consortium where metabolic interdependence and environmental adaptation contribute to the stability and persistence of a dysbiotic biofilm. Thus, *E. saphenum* should be positioned as a marker of anaerobic dysbiosis with a possible, but unproven, accessory contribution to polymicrobial biofilm stability.

### 4.1. Microbial outcomes

Studies generally indicate an increased abundance of *E. saphenum* in periodontitis compared with periodontal health, specifically in stages III–IV periodontitis [17,18]. In support of this severity-related pattern, Lafaurie et al. [18] reported an unadjusted association estimate between the presence of *E. saphenum* and Stage III–IV periodontitis, supporting its interpretation as a severity-associated microbial marker rather than evidence of causality. *E. saphenum* is more frequently observed within a broader community-level transition where subgingival ecosystems shift from a facultative, health-associated microbiota to biofilms dominated by obligate anaerobes as inflammation intensifies and pockets deepen [17,25–28]. In periodontal health, *Streptococcus* spp. act as early colonisers and help maintain microbial homeostasis, partly through antimicrobial activity [27,36]. In contrast, HOMIM profiling in aggressive periodontitis has demonstrated a significantly higher prevalence of *Streptococcus oralis, Streptococcus australis*, and *Streptococcus cristatus* in control samples, whereas diseased sites exhibited restricted *streptococcal* clusters within communities predominantly composed of anaerobes [28]. Pocket deepening reduces oxygen tension and redox potential, favouring obligate anaerobes and late colonisers, including *F. alocis* and *Peptostreptococcaceae* spp. in stage III–IV lesions [17]. Overall, *E. saphenum* may be associated with dysbiotic anaerobic communities observed in inflamed periodontal sites.

### 4.2. Sample type (biofilm vs saliva)

The included studies used either subgingival biofilm or saliva to assess *E. saphenum*. Differences in sample source may affect *E. saphenum* findings. Subgingival biofilm was sampled directly from periodontal pockets using sterile paper points [16–18,29] or sterile curettes [27]. Higher detection or abundance of *E. saphenum* in diseased pockets relative to health was observed [27,28]. Additionally, subgingival biofilm analysis further linked the microbiome, including *E. saphenum*, to increasing PPD and BOP [29]. Within included studies, *E. saphenum* tended to show higher abundance in more severe periodontitis and was among the taxa that characterised stage III–IV periodontitis [17,18].

Two studies used salivary samples to investigate *E. saphenum* [25,26]. Lundmark et al. (2019) analysed stimulated saliva and revealed that *E. saphenum* was among the taxa most strongly enriched in periodontitis, along with *T. forsythia*, *Fretibacterium* spp., and *F. nucleatum* [26]. Later, Lee et al. (2025) extended these observations using unstimulated saliva and emphasised that *E. saphenum* might act as a transitional or early dysbiosis indicator rather than a late-stage species [25]. In general, *E. saphenum* is associated with the overall dysbiotic profile of the salivary microbiome during periodontal inflammation. However, neither study assessed correlations between salivary *E. saphenum* and clinical periodontal parameters such as PPD or BOP.

Subgingival biofilm and saliva may capture different aspects of the oral ecosystem. Studies that used biofilm samples, rather than saliva, correlated *E. saphenum* with clinical periodontal parameters, including PPD and BOP [27,29]. Subgingival biofilm samples are more likely to capture the anaerobic niche in which *E. saphenum* has been detected and may better reflect site-specific microbial signals from deep periodontal pockets. This supports interpreting *E. saphenum* primarily as an ecological signal of the anaerobic subgingival pocket niche, rather than as a standalone pathogen. On the other hand, using saliva as the study sample presents several significant advantages, including being non-invasive, easily collected, and reflecting the entire microbial environment of the mouth, allowing for a broad assessment of oral bacterial communities. However, saliva introduces several limitations, such as pooling bacteria from multiple oral surfaces and diluting site-specific signals, especially *E. saphenum* that inhabits subgingival pockets. Additionally, variability in saliva collection methods, flow rate, and circadian timing can influence microbial abundance profiles, complicating comparisons between studies [37,38]. In addition, pooled subgingival samples may improve patient-level detection but can obscure site-specific microbial–clinical association, whereas site-specific sampling better reflects local pocket conditions but may increase variability [18,27,28].

### 4.3. Molecular detection methods for *E. saphenum* in periodontitis

Four analytical approaches were used for *E. saphenum* detection, which are species‑specific qPCR, 16S rRNA amplicon sequencing, HOMIM, and checkerboard DNA–DNA hybridisation. These methods differ in sensitivity, taxonomic resolution, and dependence on microbial targets [39–41]. The first method, species‑targeted qPCR on subgingival biofilm, is the most sensitive technique due to its ability to detect low-abundance targets that may be under-represented or missed by amplicon sequencing. However, qPCR measures only the targeted organisms and can miss unexpected strain diversity [16,18]. The second method, 16S rRNA amplicon sequencing, offers an open view of the oral community. In both subgingival biofilm and saliva samples, *E. saphenum* was detected in periodontitis [17,25,26,42]. Third, studies using HOMIM microarrays found that the *E. saphenum* signal increased with greater mean pocket depth, supporting its disease-related relevance [28,29]. However, arrays cannot discover species absent from the panel due to the limitation of the probes. Furthermore, closely related species may produce overlapping signals through cross-hybridisation, and the platform’s dynamic range is narrower than that of qPCRs, so low-level signals and small effect sizes are often not detected. The fourth method, the checkerboard DNA–DNA hybridisation, enables high-throughput biofilm testing using whole-genome probes [27]. This approach can detect and quantify *E. saphenum* across study groups, despite its low sensitivity at low abundance and inability to detect taxa outside the probe panel. Overall, these methodological differences may partly account for the observed patterns of *E. saphenum* detection across studies.

### 4.4. Justification of no meta-analysis

A quantitative meta-analysis was not undertaken due to substantial clinical and methodological heterogeneity among the included studies. Specifically, studies differed in the biological matrix analysed (subgingival biofilm vs saliva), detection platforms employed (qPCR, 16S rRNA sequencing, HOMIM, and checkerboard DNA–DNA hybridisation), and case definitions used to classify periodontitis and disease severity (the 1999 AAP framework versus the 2018 EFP/AAP staging system). In addition, outcome reporting varied between prevalence, relative abundance, and correlation with clinical parameters, limiting effect-size harmonisation. Given these differences, a qualitative synthesis was considered the most appropriate to summarise the available evidence without introducing misleading quantitative estimates.

### 4.5. Quality assessment, risk of bias, and certainty of evidence

Methodological quality appraisal using the AXIS tool indicated that four studies were rated as high quality and four as medium quality. The most frequently identified limitations were inadequate sample-size justification, unclear sampling frames, and incomplete reporting regarding selection bias and non-response. Nevertheless, outcome definitions and laboratory detection methods were generally appropriate, clearly described, and sufficiently detailed to support reproducibility.

Using the GRADE framework, the overall certainty of evidence for the association between *E. saphenum* and periodontitis was rated as low. This rating reflects the observational and predominantly cross-sectional nature of the included studies, which limited causal inference because of potential residual confounding and lack of temporality. Although the direction of findings was generally positive, statistical significance and severity-specific findings were not uniform. The unadjusted association estimate reported by Lafaurie et al. [18] was considered supportive of the direction and magnitude of the association, but insufficient for formal GRADE upgrading. Thus, no formal upgrade was applied because this estimate was unadjusted and derived from a single observational study; severity-specific evidence was limited, quantitative pooling was not possible, and the overall evidence remained methodologically heterogeneous and unable to establish temporality. Overall, the current body of observational evidence supports a low-certainty association between *E. saphenum* and periodontitis, with higher enrichment observed in advanced disease. Nevertheless, these findings should be interpreted cautiously in the absence of longitudinal or interventional studies capable of establishing temporality or causal relevance.

### 4.6. Limitations and research suggestions

Although the included studies represented several geographic regions, the current evidence base remains limited by the predominance of cross-sectional study designs, relatively small sample sizes, and under-representation of diverse ethnic and geographic populations. These factors restrict causal inference and may limit generalisability. In addition, variability in laboratory platforms, sampling strategies, and outcome reporting contributes to between-study heterogeneity. Greater standardisation in microbial detection methods and reporting frameworks would improve comparability and strengthen future syntheses.

Another important limitation is that major oral-microbiome confounders were not consistently controlled across the included studies. Factors such as oral hygiene status, diet, plaque accumulation, and sampling conditions may influence microbial profiles. Therefore, the observed association between *E. saphenum* and periodontitis may partly reflect uncontrolled confounding rather than an independent microbial effect. These sources of heterogeneity may have contributed to the mixed statistical significance observed across studies and limited the strength of any claim regarding a consistent association. These limitations also leave reverse causation unresolved, as *E. saphenum* may increase because periodontal breakdown creates a favourable anaerobic, nutrient-rich niche rather than because the organism independently drives disease progression.

Future studies should prioritise longitudinal designs to determine whether increased *E. saphenum* precedes periodontal progression or emerges after pocket deepening and ecological shifts within established dysbiosis. Interventional studies evaluating changes in *E. saphenum* levels following periodontal therapy, and correlating microbial shifts with clinical outcomes, would further help clarify its ecological and functional relevance. In addition, mechanistic investigations, including isolate‑level genomics and *in vitro* functional models, are needed to characterise potential virulence attributes such as immunostimulatory pathways, stress adaptation, and interspecies interactions within polymicrobial biofilms. Future studies should also evaluate co-occurrence and network relationships between *E. saphenum* and established periodontal taxa to determine whether it represents a passive marker of anaerobic dysbiosis or a functionally relevant member of pathogenic microbial consortia.

## 5. Conclusion

In conclusion, the available observational evidence provides preliminary support for an association between *E. saphenum* and periodontitis, with higher detection and abundance reported in advanced stages of disease. Although *E. saphenum* appears to be a recurrently reported feature of periodontal dysbiosis, the current evidence does not permit causal inference. Well-designed longitudinal and interventional studies are required to clarify whether *E. saphenum* contributes functionally to periodontal disease progression or represents an ecological consequence of established disease.

## Supporting information

S1 File Dataset supporting the systematic review. (XLSX)

S2 File Supplementary dataset. (XLSX)

S3 File PRISMA 2020 checklist. (DOCX)
