# Supplementary material for: Association between Eubacterium saphenum and periodontitis: A systematic review
Source: PLoS One. 2026 Sep 2;21(9):e0357110. doi: 10.1371/journal.pone.0357110 (PMC13537536; doi:10.1371/journal.pone.0357110)
Supplement: Supplementary file 2 [file pone.0357110.s005.docx]

**Response to Reviewers**

Dear Editor and reviewer/s

We sincerely thank the reviewers for their thoughtful and constructive comments. We have carefully considered all suggestions and revised the manuscript accordingly. We believe that these revisions have substantially improved the clarity, methodological rigour, and overall quality of the manuscript. A detailed point-by-point response is provided below.

|  | **Reviewer #1** | **Response** |
| --- | --- | --- |
| 1 | The manuscript repeatedly refers to a ‘consistent association’ between *E. saphenum* and periodontitis. However, Table 1 shows mixed findings, with several studies reporting no significant association. The conclusions should reflect a trend or emerging association rather than a consistent one. | We agree with the reviewer that the term "consistent association" overstated the available evidence. The manuscript has been revised throughout to describe the findings as a low-certainty positive directional association, while acknowledging mixed statistical findings and methodological heterogeneity.  **Location in revised manuscript.** Abstract, p. 2; Results Section 3.4, p. 9; Results/GRADE Section 3.5, p. 10; Table 4, p. 17; Discussion, pp. 17-22; Conclusion, p. 23. |
| 2 | The decision not to perform a meta-analysis is understandable given heterogeneity in sample types, detection methods, and case definitions. However, the justification provided remains limited.  The authors should either strengthen the justification (e.g., explicitly discuss incompatibility of outcome metrics), or provide a semi-quantitative synthesis (e.g., proportion of studies showing positive associations, direction of effects). | In response to the reviewer's suggestion, we added a structured semi-quantitative synthesis describing:   - number of studies reporting positive associations, - number reporting severity-related findings, - sample type distribution, - nature of microbial outcomes reported.   **Location in revised manuscript.** Methods Section 2.4, pp. 5-6; Results Section 3.4, p. 9; Discussion, Justification for not conducting a meta-analysis, p. 21.  **Revised text:**  A quantitative meta-analysis was not performed because the included studies showed substantial clinical and methodological heterogeneity in sample matrix, microbial detection platform, periodontal case definition, disease-severity classification, and microbial outcome reporting.  A structured qualitative synthesis was undertaken to summarise the direction and type of evidence across the included studies. At the study level, all eight included studies reported findings in a positive qualitative direction for *E. saphenum* in relation to periodontitis, advanced disease, or periodontal disease-associated microbial profiles. |
| 3 | GRADE assessment likely overestimated The upgrade to “moderate certainty” appears optimistic given exclusively observational (mostly cross-sectional) data, small sample sizes and methodological heterogeneity. A more cautious interpretation (e.g., low certainty) should be considered, or the rationale for upgrading should be expanded. | We thank the reviewer for this important methodological comment and agree with the recommendation. The GRADE assessment was revised using a more conservative domain-by-domain approach. Accordingly, the certainty of evidence was downgraded from moderate to low, reflecting the observational and predominantly cross-sectional nature of the included studies, modest sample sizes, methodological heterogeneity, limited adjusted estimates across the evidence base and the absence of evidence establishing temporality. The unadjusted odd ratio (OR = 4.85) was retained as supportive evidence but was not used as a for formal upgrading criterion.  **Location in revised manuscript.** Methods Section 2.4, pp. 5-6; Results Section 3.5, p. 9-10; Table 4, p. 17; Discussion Section 4.5, p. 21-22.  **Revised text:**  Potential upgrading factors, including large estimate and severity-related patterns, were considered descriptively. However, because cross-study agreement could only be assessed qualitatively across heterogeneous methodologies, consistency was not used as a formal upgrading criterion.  Therefore, the overall certainty of evidence was conservatively rated as low for the association between *E. saphenum* and periodontitis. |
| 4 | While detection methods are described, their limitations are not critically discussed. Important sources of bias include targeted vs untargeted detection approaches, taxonomic resolution issues, pooling vs site-specific sampling and variability in sequencing platforms.  A deeper methodological critique is needed to contextualize the findings. | We thank the reviewer for this important comment. We expanded the methodological critique to explain how detection platform, bioinformatic pipelines, and sampling strategy may influence detection and comparability. We now clarify that qPCR copy number, HOMIM/checkerboard signal intensity, and sequencing-derived relative abundance should not be interpreted as directly equivalent outcomes.  **Location in revised manuscript.** Results Section 3.3, pp. 8-9; Discussion Section 4.2, pp. 19-20; Discussion Section 4.3, pp. 20-21.  **Revised text:**  These platform differences may introduce detection bias because targeted methods, such as qPCR, HOMIM and checkerboard DNA-DNA hybridisation, depend on predefined primers or probes, whereas 16S rRNA sequencing is affected by primer choice, amplified region, sequencing depth, database selection and bioinformatic pipeline.  Consequently, qPCR copy number, HOMIM/checkerboard signal intensity and sequence-derived relative abundance should not be interpreted as directly equivalent outcomes. |
| 5 | The manuscript appropriately avoids causal claims, but the discussion remains superficial regarding: reverse causation, ecological shifts vs pathogenic role, whether *E. saphenum* is a marker or contributor. The authors should strengthen this section to provide a clearer conceptual positioning. | We thank the reviewer for this valuable comment. We strengthened the conceptual positioning throughout the Discussion and Limitations. *E. saphenum* is now framed primarily as a severity-associated ecological marker of anaerobic dysbiosis, while a contributory role within polymicrobial anaerobic consortia is described as biologically plausible but unproven. Reverse causation is explicitly acknowledged.  **Location in revised manuscript.** Introduction, pp. 3-4; Discussion opening, pp. 17-18; Limitations and Research Suggestions, p. 22; Conclusion, p. 23.  **Revised text:**  Rather than representing a standalone pathogen, *E. saphenum* is best conceptualised as a severity-associated microbial signal within polymicrobial periodontal dysbiosis.  These limitations also leave reverse causation unresolved, as *E. saphenum* may increase because periodontal breakdown creates a favourable anaerobic, nutrient-rich niche rather than because the organism independently drives disease progression. |
| 6 | Minor comments. The manuscript is generally understandable but contains several grammatical and typographical errors (e.g., “Then certainty of evidence…”, “dynamicecological”, missing verbs). A thorough language revision by a native English speaker is recommended. | We revised the manuscript for grammar, punctuation, spacing, spelling, and scientific clarity. We corrected duplicated punctuation, duplicated citations, awkward wording, and typographical errors throughout the manuscript.  **Location in revised manuscript.** Throughout the revised manuscript, especially Introduction, Methods, Results, and Discussion. |
| 7 | The introduction is informative but somewhat broad. Consider sharpening the research gap and more clearly justifying the focus on *E. saphenum* specifically. | We sharpened the Introduction by clarifying that the research gap is whether *E. saphenum* is a reproducible severity-associated component of periodontal dysbiosis, not whether it is a proven causal pathogen. The rationale for focusing on *E. saphenum* was strengthened by linking its anaerobic niche, repeated molecular detection, and fragmented evidence base.  **Location in revised manuscript.** Introduction, pp. 3-4.  **Revised text:**  Therefore, the key gap is whether the repeated detection of *E. saphenum* reflects a reproducible severity-associated component of periodontal dysbiosis, particularly in advanced periodontitis, and how this taxon should be interpreted within the broader polymicrobial dysbiosis framework. |
| 8 | Results section is overly descriptive. The addition of simple quantitative summaries (e.g., number of studies supporting the association) would improve clarity. | We added study-level summaries to Section 3.4, including the number of studies showing a positive qualitative direction, the number using subgingival biofilm versus saliva, and the number providing severity-specific evidence.  **Location in revised manuscript.** Results Section 3.4, p. 9.  **Revised text:**  Six studies analysed subgingival biofilm and two analysed saliva. All eight included studies contributed findings in a positive qualitative direction for *E. saphenum* in relation to periodontitis, advanced disease, or periodontal disease-associated microbial profiles. However, we clarified that the studies supported this pattern in different ways, including increased detection or abundance, clinical correlations, microbial-community associations, and qPCR detection evidence. |
| 9 | Tables could be more informative Table 1 is useful but could be enhanced by including effect sizes (if available), statistical significance indicators. | We expanded Table 1 by adding a column for quantitative/statistical indicators, including available ORs, confidence intervals, p-values, correlation coefficients, detection metrics, and other study-specific statistics.  **Location in revised manuscript.** Table 1, pp. 11-13.  **Revised text:**  A new column titled ‘Quantitative/statistical finding’ was added to Table 1 to allow rapid comparison of available study-level evidence. |
|  | **Reviewer #2** | **Response** |
| 1 | Minor correction. Abstract: please check the correct typing of *Eubactrium saphenum* as its currently written Eubatcriumsaphenum, non-italicized, and without space. Same applies to *E. saphenum*. | Thank you for your note. We corrected spelling, spacing, and bacterial nomenclature formatting throughout the manuscript. Genus and species names were italicized where appropriate, including *Eubacterium saphenum* and *E. saphenum* in the Abstract, main text, tables, and figure captions.  **Location in revised manuscript.** Title and Abstract, p. 2; throughout manuscript and tables.  **Revised text:**  The bacterial name is now presented consistently as *Eubacterium saphenum* at first use and *E. saphenum* thereafter, with appropriate italic formatting. |
| 2 | Minor correction. In abstract, I suggest clarifying the classification system in the phrase “Studies applying the 2018 classification system consistently reported greater..” by stating 2018 periodontal classification system or referencing the Classification of Periodontal and Peri-Implant Diseases and Conditions. | Thank you for your note, we agree. The abstract has been revised to specify the 2018 Periodontal Classification system. We have also checked the manuscript and tables to ensure that the classification terminology is used consistently throughout.  **Location in revised manuscript.** Abstract, p. 2; Table 1, pp. 11-13.  **Revised text:**  Among studies applying the 2018 periodontal classification system and reporting stage-specific analyses, higher detection or enrichment of *E. saphenum* was generally observed in advanced disease (Stage III–IV). |
| 3 | Adding justification. Axis tool was used for quality assessment per section 2.4. Justification needed for not utilizing other assessment tools such as The Joanna Briggs Institute (JBI). I referenced Downes MJ et al. to evaluate different assessment tools, specifically to address Cui et al. 2019, Castillo et al. 2023, Marchensan et al. 2015 cited in the paper.  Ma LL, Wang YY, Yang ZH, Huang D, Weng H, Zeng XT. Methodological quality (risk of bias) assessment tools for primary and secondary medical studies: what are they and which is better? Mil Med Res. 2020 Feb 29;7(1):7. doi: 10.1186/s40779-020-00238-8. PMID: 32111253; PMCID: PMC7049186. | We thank the reviewer for this important comment. Following the reviewer’s recommendation and the methodological guidance of Downes et al. and Ma et al., we have clarified the rationale for selecting the AXIS tool in Section 2.4. AXIS was chosen because the included studies were observational and predominantly cross-sectional, and the review required assessment of both methodological quality and reporting transparency. While the Joanna Briggs Institute (JBI) checklist is also appropriate for analytical cross-sectional studies, it primarily focuses on risk-of-bias domains such as exposure and outcome measurement, confounding, and statistical analysis. In contrast, AXIS provides a broader 20-item framework that additionally evaluates sample-size justification, sampling strategy, representativeness, non-response, study limitations, ethical approval, and potential conflicts of interest. Therefore, AXIS was considered the most suitable tool for providing a comprehensive and consistent appraisal of the heterogeneous microbiome studies included in this review.  **Location in revised manuscript.** Methods Section 2.4, pp. 5-7.  **Revised text:**  The AXIS tool was selected because the included studies were observational and predominantly cross-sectional in design, involving comparisons of microbial detection or relative abundance between periodontal health and periodontitis groups. Although the Joanna Briggs Institute checklist is also appropriate for analytical cross-sectional studies, AXIS was chosen because it provides a broader 20-item framework for evaluating both methodological quality and reporting transparency, including sample-size justification, sampling frame, representativeness, non-response, limitations, ethical approval, and potential conflicts of interest. This approach is consistent with methodological guidance recommending that appraisal tools should be selected according to the design and methodological characteristics of the included studies [22]. |
| 4a | If the one study rated as high quality in section 4.5, Lafaurie et al. 2023, is what is meant by strong in association and magnitude as reflected in Odds ratio [12] table 4, please make that it more clear in section 4.5 | We thank the reviewer for this important clarification. We agree that the previous wording in Section 4.5 could be misinterpreted as indicating that only one study was rated as high quality. We have revised Section 4.5 to clarify that the AXIS methodological quality appraisal identified four high-quality studies and four medium-quality studies, as shown in Table 3.  We also clarified that the reference to Lafaurie et al. 2023 [18] in relation to GRADE refers specifically to the magnitude of association reported in that study, reflected by the odds ratio of 4.85, rather than to the number of studies rated as high quality. This distinction has now been made explicit in Section 4.5. We also revised the Table 4 footnote to state “odds ratio” correctly and to clarify that the estimate refers to the association reported by Lafaurie et al. 2023.  **Location in revised manuscript.** Results Section 3.5, pp. 9-10; Table 3, p. 16; Table 4, p. 17; Discussion Section 4.5, pp. 21-22.  **Revised text:**  The overall scores ranged from medium to high quality. According to the predefined criteria, four studies were of high quality, and the remaining four studies were rated as medium quality. |
| 4b | Minor correction: typo in the odds ratio footnote in Table 4. | We thank the reviewer for noting this typographical error. The footnote in Table 4 has been corrected to state ‘odds ratio’ and to identify the estimate as unadjusted.  **Location in revised manuscript.** Table 4, p. 17.  **Revised text:**  Unadjusted odds ratio / bivariate estimate reported by Lafaurie et al. 2023 [18] for the association between the presence of *E. saphenum* and Stage III–IV periodontitis compared with health (95% CI 1.99–11.7). It was interpreted as supportive of the direction and magnitude of association. |
| 5 | In 2.2 Search Strategy, Google Scholar was among the search. Using the author Google Scholar search criteria, I manually searched for khemwong et al 2018. Title: *Eubacterium saphenum* as a Novel Bacterial Biomarker for Periodontitis Screening, I was not able to find the paper in the first 200 paper or first 20 pages. I used the keyword "*Eubacterium saphenum*" OR "*E. saphenum*" OR "*E. saphenus*" periodontitis OR "periodontal disease". However, Khemwong et al 2018 is available in Google Scholar. Please provide insight into the sensitivity of the search as a potential relevant study may be beneficial to the study and was not cited. | We sincerely thank the reviewer for this careful observation and for highlighting Khemwong et al. (2018). This study was identified during the original search process and assessed at the full-text eligibility stage. However, it was excluded according to the predefined eligibility criteria because periodontitis was defined as the presence of at least one tooth with PPD ≥4 mm, which did not correspond to a recognised periodontal classification system or a clearly defined diagnosis incorporating clinical attachment loss, radiographic bone loss, and disease extent or severity. Consequently, the study was excluded under the category “unclear definition of periodontitis” in the PRISMA flowchart. Importantly, because the study was successfully identified during the search process, its exclusion reflects the application of the eligibility criteria rather than a limitation in search sensitivity.  **Location in revised manuscript.** Eligibility Criteria, pp. 4-5; Results Section 3.1, p. 7; PRISMA flowchart, Fig. 1. |
| 6 | In section 3.5, there was an introduction to a new category: high high-quality. Please correct if typo error. | We thank the reviewer for noting this typographical error. The term “high high-quality” in Section 3.5 was corrected.  **Location in revised manuscript.** Result section 3.5 pp. 9-10.  **Revised text:**  First, the risk of bias raised some concerns because four studies were rated as medium quality and four as high quality, with common limitations in sample-size justification and sampling frames. |
|  | **Reviewer #3** | **Response** |
| 1 | The manuscript is generally well written, with only a few minor issues related to formatting and style, and the methodological approach appears appropriate. However, the scientific contribution and the amount of knowledge generated from the research question seem somewhat limited. This concern is also reflected in the relatively scarce bibliography supporting the study. | We appreciate this observation. The review was intentionally focused on *E. saphenum* because it is a non-classical anaerobic taxon that has been repeatedly detected in periodontitis, particularly in advanced disease. This raises an important question: whether the repeated detection of *E. saphenum* reflects a reproducible severity-associated component of periodontal dysbiosis and how this taxon should be interpreted within the broader polymicrobial dysbiosis framework. We have revised the Introduction and Discussion to clarify this rationale. The small number of eligible studies is now acknowledged as a limitation of the evidence base, rather than presented as the main rationale for the review.  **Location in revised manuscript.** Introduction, pp. 3-4; Discussion opening and Sections 4.1–4.6, pp.17-22; Conclusion. |
|  | I would suggest incorporating additional bacterial species of major relevance in periodontology, as this could broaden the scope of the analysis, provide greater scientific depth, and strengthen the overall contribution of the manuscript. | We also thank the reviewer for this insightful comment. We agree that a broader review including multiple established periodontal bacterial species could provide a more comprehensive overview of the periodontal microbiome. However, the scope of the present systematic review was defined a priori to address a specific and underexplored evidence gap: the association between *Eubacterium saphenum* and periodontitis. This question was predefined in the PECO framework and prospectively registered in PROSPERO. Incorporating additional bacterial species would require a substantially different review question, search strategy, eligibility criteria, data extraction framework, and synthesis plan, representing a post hoc deviation from the registered protocol.  In response to the valuable suggestion, we have revised the Introduction and Discussion to contextualise *E. saphenum* within the broader polymicrobial dysbiosis model of periodontitis. We believe these additions provide important biological context while maintaining the predefined focus and methodological integrity of the review.    **Location in revised manuscript.** Introduction, p. 3; Discussion/Limitation and Future Research, p. 22.  **Revised text:**  Although established periodontal pathobionts, including *Porphyromonas gingivalis,* *Tannerella forsythia*, *Treponema denticola*, *Aggregatibacter actinomycetemcomitans,* *Fusobacterium nucleatum*, and *Filifactor alocis*, have been widely implicated in periodontitis, their detection alone does not fully explain inter-individual variation in disease severity or progression, supporting a community-level dysbiosis model of disease.  Future studies should also evaluate co-occurrence and network relationships between *E. saphenum* and established periodontal taxa to determine whether it represents a passive marker of anaerobic dysbiosis or a functionally relevant member of pathogenic microbial consortia. |
|  | **Reviewer #4** | **Response** |
| Introduction | some sentences would benefit from grammatical revision and improved clarity. For example, the phrase “Contemporary models emphasise on polymicrobial synergy and dysbiosis” should be revised for grammatical accuracy. Similarly, the sentence describing *E. saphenum* as “an obligate anaerobes, Gram-positive species” contains grammatical inconsistencies.  The introduction also states that *E. saphenum* “may serve as biomarkers of disease severity or participate in pathogenic consortia,” which is acceptable as a hypothesis; however, maintaining cautious language throughout the manuscript would improve consistency with the observational nature of the included studies. | We sincerely thank the reviewer for the positive assessment of the Introduction and for the constructive suggestions regarding language precision and consistency of interpretation. We revised the Introduction to improve grammar and cautious interpretation. We removed wording that could imply causality and framed *E. saphenum* as a potential marker or associated member of dysbiotic communities rather than a confirmed pathogenic driver.  **Location in revised manuscript.** Introduction, pp. 3-4.  **Revised text:**  Contemporary models therefore emphasise polymicrobial synergy and dysbiosis. *E. saphenum* is a Gram-positive obligate anaerobe, |
| Methodology 1 | The methodology section is generally well organized and clearly describes the PECO framework, eligibility criteria, databases searched, and quality assessment methods. However, several clarifications would improve transparency and reproducibility.  The search strategy would benefit from additional detail. Although the databases and search terms are reported, the manuscript does not specify the number of records retrieved from each individual database before deduplication. It would be helpful if the authors included this information either within the PRISMA flowchart or in a supplementary table. Specifically, the number of articles identified from PubMed/MEDLINE, Scopus, Web of Science, Cochrane Library, and Google Scholar should be individually reported. This addition would improve transparency regarding study identification and screening. | We agree that reporting database-specific yields before deduplication improves transparency. Accordingly, we have added a table to report the pre-deduplication yields from each searched database/source before duplicate removal. This table is included as a supplementary file to the manuscript.  **Location in revised manuscript:** In supplementary file.  **Revised text:**  Before deduplication, database searches identified 753 records. PubMed/MEDLINE (n= 283); Scopus (n= 88); Web of Science Core Collection (n= 163); Cochrane Library (n=19); Google Scholar: (n=200). After removal of 291 duplicate records using Zotero and Rayyan, 462 unique records remained for title and abstract screening. |
| Methodology 2 | The justification for not conducting a meta-analysis is appropriately explained based on heterogeneity in sample type, detection methods, and case definitions. Nevertheless, because the manuscript repeatedly refers to “consistent findings,” it may be useful to clarify that the consistency observed was primarily qualitative across studies using different methodologies. | Thank you for this helpful comment. We have revised the manuscript to clarify that the observed consistency refers to qualitative directional agreement across studies using heterogeneous sample types, detection platforms, periodontal case definitions, and outcome metrics, rather than statistical homogeneity or quantitatively comparable effect sizes.  **Location in revised manuscript.** Methods Section 2.4, pp. 5-6; Results Sections 3.4-3.5, pp. 9-10; Table 4, p. 17; Discussion pp. 17-22; Conclusion, p. 23.  **Revised text:**  Because cross-study agreement could only be assessed qualitatively across heterogeneous methodologies, consistency was not used as a formal upgrading criterion.  Cross-study consistency was qualitative rather than quantitative, and no included study established temporality. |
| Methodology 3 | The section describing quality assessment and certainty of evidence is adequately structured. However, the sentence “four studies of high high-quality” contains a typographical duplication that should be corrected. | We thank the reviewer for noting this typographical error. The typographical duplication was corrected.  **Location in revised manuscript.** Results Section 3.5, pp. 9-10.  **Revised text:**  Four studies were of high quality, and the remaining four studies were rated as medium quality. |
| Discussion | several sentences throughout the discussion would benefit from improved wording and grammatical revision. For example, the expression “dynamicecological changes” contains a typographical error. In addition, there are several formatting inconsistencies involving the presentation of *E. saphenum* throughout the text.  Some statements in the discussion are more interpretive than directly supported by the included studies. For example, phrases suggesting that *E. saphenum* may “reinforce” inflammatory microbial communities should be carefully worded to remain consistent with the observational design of the included evidence. The limitations section is appropriate and acknowledges the predominance of cross-sectional studies, small sample sizes, and methodological heterogeneity. This strengthens the overall balance of the discussion. | We thank the reviewer for the constructive feedback. We have carefully revised the Discussion to improve grammar, clarity, and formatting. The typographical error “dynamicecological changes” has been corrected to “These observations are consistent with ecological succession in the subgingival environment as disease progresses, rather than evidence that a single organism independently drives tissue destruction”, and all occurrences of *E. saphenum* have been standardized.  Interpretive statements suggesting causality have been reworded to reflect associations observed in the included cross-sectional studies. For example, phrases implying that *E. saphenum* “reinforces” inflammatory communities are now presented as co-occurrence within dysbiotic microbial ecosystems. Long or unclear sentences were simplified to enhance readability, and the limitations section has been refined to emphasize the need for longitudinal and mechanistic studies to determine temporality or causal roles.  **Location in revised manuscript.** Discussion, pp. 17-22.  **Revised text:**  Rather than representing a standalone pathogen, *E. saphenum* is best conceptualised as a severity-associated microbial signal within polymicrobial periodontal dysbiosis.  Although the specific virulence mechanisms of *E. saphenum* remain unclear, its recurrent detection alongside established late colonisers suggests that it may co-occur within cooperative anaerobic consortia.  These observations are consistent with ecological succession in the subgingival environment as disease progresses, rather than evidence that a single organism independently drives tissue destruction |
| Conclusions | The conclusions are generally aligned with the presented findings and appropriately acknowledge that the available evidence does not establish causality. The recommendation for future longitudinal and interventional studies is justified and consistent with the limitations identified in the review. | We thank the reviewer for their positive assessment of the Conclusions section. We appreciate the acknowledgment that the conclusions accurately reflect the findings and justify the recommendation for future longitudinal and interventional studies.  **Location in revised manuscript.** Conclusion, p. 23.  **Revised text:**  The current evidence does not permit causal inference. Well-designed longitudinal and interventional studies are required to clarify whether *E. saphenum* contributes functionally to periodontal disease progression or represents an ecological consequence of established disease. |
| Grammar | The manuscript would benefit from careful English-language editing to improve readability and grammatical consistency. Examples identified in the current version include:  • “Then certainty of evidence was rated as moderate” should be revised to “The certainty of evidence was rated as moderate.” | We thank the reviewer for this helpful comment. We have carefully edited the manuscript to improve English-language clarity, readability, and grammatical consistency. Specifically, the sentence “Then certainty of evidence…” has been revised to “The certainty of evidence …...” Similar grammatical and stylistic issues were also reviewed and corrected throughout the manuscript.  **Location in revised manuscript.** Throughout manuscript; especially Abstract, Introduction, Results Section 3.5, and Discussion.  **Revised text:**  The certainty of evidence was rated as low owing to the observational and predominantly cross-sectional design of the evidence, methodological heterogeneity, and lack of temporality. |
|  | • “*E. saphenum* is an obligate anaerobes” should be revised to “*E. saphenum* is an obligate anaerobe.” | We thank the reviewer for noting this grammatical inconsistency. The phrase “*E. saphenum* is an obligate anaerobe” has been corrected to “*E. saphenum* is a Gram-positive obligate anaerobe.” This revision improves grammatical accuracy and clarity  **Location in revised manuscript.** Introduction section p. 3.  **Revised text:**  *E. saphenum* is a Gram-positive obligate anaerobe. |
|  | • “Contemporary models emphasise on polymicrobial synergy” should be revised grammatically. | We thank the reviewer for identifying this grammatical issue. The phrase “Contemporary models emphasise on polymicrobial synergy and dysbiosis” has been revised to “Contemporary models emphasise polymicrobial synergy and dysbiosis.”  **Location in revised manuscript.** Introduction section p. 3.  **Revised text**  Contemporary models therefore emphasise polymicrobial synergy and dysbiosis. |
|  | dynamicecological changes” contains a typographical error. | We thank the reviewer for noting this typographical error. The term “dynamicecological changes” was corrected to “These observations are consistent with ecological succession in the subgingival environment as disease progresses, rather than evidence that a single organism independently drives tissue destruction”.  **Location in revised manuscript.** Discussion section p. 17.  **Revised text**  These observations are consistent with ecological succession in the subgingival environment as disease progresses, rather than evidence that a single organism independently drives tissue destruction. |
|  | • “high high-quality” contains duplicated wording. | We thank the reviewer for noting this typographical error. The term “high high-quality” in Section 3.5 was corrected.  **Location in revised manuscript.** Result section 3.5 pp. 9-10.  **Revised text**  First, the risk of bias raised some concerns because four studies were rated as medium quality and four as high quality, with common limitations in sample-size justification and sampling frames. |
|  | • Minor formatting inconsistencies involving “*E.saphenum*” versus “*E. saphenum*” should be standardized throughout the manuscript. | We thank the reviewer for identifying this formatting inconsistency. We have standardised the abbreviation throughout the manuscript as “*E. saphenum*,” with a space after the abbreviated genus initial. This correction has been applied consistently across the title, abstract, main text, tables, and figure captions. |
| OR/GRADE | the section discussing certainty of evidence and the upgrading of the GRADE assessment would benefit from greater methodologica detail regarding the reported odds ratio (OR = 4.85). The manuscript should clarify whether this estimate was obtained from an adjusted multivariable model or from a bivariate analysis only. If the OR was adjusted, the variables included in the adjustment model should be explicitly reported. If it corresponds to an unadjusted association, this limitation should be acknowledged when interpreting the magnitude of effect.  Furthermore, confidence intervals should be consistently reported alongside effect estimates. In particular, the 95% confidence interval (95% CI) corresponding to the reported odds ratio should be included to allow readers to assess the precision of the estimate. | We thank the reviewer for this comment. We clarified that the OR = 4.85 was reported by Lafaurie et al. 2023 and is an unadjusted / bivariate estimate with 95% CI 1.99-11.7. It was interpreted as supportive of the direction and magnitude of association but insufficient for formal GRADE upgrading because it was unadjusted, derived from one observational study, and did not establish temporality.  **Location in revised manuscript.** Results Section 3.5, pp. 9-10; Table 1, pp. 11-13; Table 4, p. 17; Discussion Section 4.5, pp. 21-22.  **Revised text:**  Potential upgrading factors were considered, including severity-related enrichment and the unadjusted odds ratio (OR = 4.85, 95% CI 1.99-11.7) / bivariate estimate reported by Lafaurie et al. [18]. However, no formal upgrade was applied because this estimate was unadjusted and derived from a single observational study. |
|  | **Reviewer #5** | **Response** |
| 1 | Statistical Interpretation: The manuscript mentions “moderate-to-large effect estimates (OR = 4.85)” but does not sufficiently explain the source study or context of this odds ratio. Please clarify:  • Which study reported this OR.  • Whether the OR was adjusted or unadjusted.  • Confidence intervals and statistical significance. | We thank the reviewer for requesting clarification. We clarified that the OR = 4.85 was reported by Lafaurie et al. 2023 and represents an unadjusted association estimate for Stage III-IV periodontitis versus periodontal health. We added the 95% CI (1.99-11.7) and clarified that the estimate was not used as a formal GRADE upgrading criterion.  **Location in revised manuscript.** Results Section 3.5, pp. 9-10; Table 1, pp. 11-13; Table 4, p. 17.  **Revised text:**  Stage III-IV vs health: OR = 4.85, 95% CI 1.99-11.7. The OR from Lafaurie et al., 2023 [18] is the unadjusted estimate. |
|  | Minor Comments: |  |
| 1 | Ensure consistency in bacterial nomenclature formatting throughout the manuscript (italicization of species names). | Thank you for pointing this out. We performed a full nomenclature check and italicized genus/species names where appropriate throughout the manuscript, tables, and captions.  **Location in revised manuscript.** Throughout manuscript; Abstract p. 2; Tables 1-4, pp. 11-17. |
| 2 | Some references require formatting consistency according to journal style. | We reviewed the reference list for consistency in punctuation, journal names, volume/issue formatting, page ranges, and capitalization according to the journal style.  **Location in revised manuscript.** Introduction, p. 3; References, pp. 23-27.  **Revised text:**  Reference formatting has been standardised throughout the revised manuscript. |
| 3 | Table formatting can be improved for readability, particularly Tables 2-4. | We reformatted Tables 2-4 to improve readability by standardising font size, alignment, spacing, headings, abbreviations, and footnotes. We also clarified Table 4 wording to avoid overstatement of qualitative consistency.  **Location in revised manuscript.** Tables 2-4, pp. 13-17.  **Revised text:**  Table 4 now distinguishes qualitative directional agreement from quantitative homogeneity and states that the certainty of evidence remains low. |
| 4 | The PRISMA flow diagram should be provided in higher resolution. | We thank the reviewer for pointing this out. The PRISMA flow diagram has been provided with a higher-resolution version.  **Location in revised manuscript.** Figure 1, PRISMA flowchart. |
| 5 | Consider simplifying lengthy discussion paragraphs for improved readability. | We thank the reviewer for this helpful comment. We revised the Discussion to improve readability by shortening lengthy sentences, splitting long paragraphs and deleting repeated statements. The expanded scientific content was retained but presented in a more concise and readable form.  **Location in revised manuscript.** Discussion, pp. 17-22.  **Revised text:**  The revised Discussion presents *E. saphenum* as a severity-associated microbial signal within polymicrobial periodontal dysbiosis, while avoiding unsupported causal claims. |
|  | **Reviewer #6** | **Response** |
| 1 | The biggest limitation is that all the evidence is observational and cross-sectional, so the association is strong but by definition, is not non-causal. You cannot determine whether *E. saphenum* contributes to disease or is simply a byproduct of dysbiosis. | We thank the reviewer for this important comment. We agree that the observational and predominantly cross-sectional evidence cannot establish temporality or causality. We have therefore revised the manuscript to interpret *E. saphenum* as a severity-associated marker of periodontal dysbiosis, rather than a confirmed causal pathogen.  **Location in revised manuscript.** Abstract, p. 2; Introduction, p. 3; Discussion, pp. 17-22; Conclusion, p. 23.  **Revised text:**  Findings in this review were interpreted as associations rather than evidence of causality. |
| 2 | The authors did not appear to fully analyze subgroup or structured comparisons to understand sources of heterogeneity. This heterogeneity may be important to explain variability in findings! | We addressed heterogeneity through a structured qualitative synthesis using predefined domains: sample type, detection platform, periodontal diagnostic classification, microbial outcome metric, and severity-specific analysis. We added text explaining that variation in diagnosis, outcomes, and detection methods limited direct comparability and justified qualitative rather than quantitative synthesis.  **Location in revised manuscript.** Methods Section 2.4, pp. 5-6; Results Sections 3.2-3.4, pp. 7-10.  **Revised text:**  Studies were compared according to five predefined domains: biological sample type, detection method, periodontal diagnostic framework, microbial outcome metric, and availability of severity-specific analysis. |
| 3 | There seemed to be some variation in how disease and microbial outcomes are defined, which may have limited comparability across studies | We thank the reviewer for this important comment. We have revised Sections 2.4, 3.2, and 3.3 to clarify that heterogeneity was addressed through a structured synthesis. We also clarified that variation in diagnostic criteria, microbial outcome definitions, and detection methods limited direct comparability across studies and therefore justified narrative synthesis rather than quantitative meta-analysis.  **Location in revised manuscript.** Methods Section 2.4, pp. 5-6; Limitations, p. 22; Conclusion, p. 23.  **Revised text:**  To reduce subjectivity in the qualitative synthesis, these domains were extracted systematically for each included study |
| 4 | Big confounders that can influence the oral microbiome are not really (or at the very least not consistently) controlled for across studies. These may have had large impacts on the oral microbiome. | We thank the reviewer for this important comment. We have revised the Limitations section to explicitly acknowledge inconsistent control of major oral-microbiome confounders, including oral hygiene status, plaque accumulation, and sampling conditions. We further clarified that these factors may partly explain the observed association between *E. saphenum* and periodontitis.  **Location in revised manuscript.** Limitations and Research Suggestions, p. 22.  **Revised text:**  Factors such as oral hygiene status, diet, plaque accumulation, and sampling conditions may influence microbial profiles. Therefore, the observed association between *E. saphenum* and periodontitis may partly reflect uncontrolled confounding rather than an independent microbial effect. |
| 5 | Minor Comments:  The relatively small number of studies limits the strength and generalizability of the conclusions. | We thank the reviewer for these comments. We agree that the small number of included studies limits the strength and generalizability of the conclusions. These limitations have been acknowledged in the manuscript, and the conclusions have been framed cautiously.  **Location in revised manuscript.** Limitations, p. 22; Conclusion, p. 23.  **Revised text:**  Despite global collaboration, the current evidence base remains limited by the predominance of cross-sectional study designs, relatively small sample sizes, and underrepresentation of diverse ethnic and geographic populations. These factors restrict causal inference and may limit generalisability. |
| 6 | The narrative approach introduces more subjectivity in the study findings. | We thank the reviewer for this comment. We agree that narrative synthesis may introduce subjectivity. To reduce subjectivity, the Methods section also clarifies that the narrative synthesis was structured using predefined domains, including sample type, detection platform, periodontal diagnostic framework, microbial outcome metric, and severity-specific analysis.  **Location in revised manuscript.** Methods Section 2.4, pp. 5–6; Results Section 3.4, p. 9; Limitations Section 4.6, p. 22  **Revised text:**  To reduce subjectivity in the qualitative synthesis, predefined domains were extracted systematically for each included study, including sample type, detection method, periodontal diagnostic framework, microbial outcome metric, and severity-specific analysis. |
|  | **Reviewer #7** | **Response** |
| 1 | The manuscript is interesting and addresses a relevant topic. I would suggest a careful English language revision throughout the text. In addition, the tables should be standardized, particularly regarding font style and formatting consistency. | We sincerely thank the reviewer for the positive evaluation of our manuscript and for the helpful suggestions to improve its clarity, presentation, and structure. The manuscript has undergone careful English language revision throughout the text. We corrected grammatical errors, improved sentence structure, standardized terminology, and refined the scientific wording to enhance readability and clarity.  **Location in revised manuscript.** Throughout manuscript; Tables 1-4, pp. 11-17.  **Revised text:**  Tables were standardised to improve readability and consistency across the manuscript. |
| 2 | In the Results section, after presenting each set of results, I also recommend citing the corresponding table in parentheses (e.g., Table 1). | We reviewed the Results section and ensured that table citations were placed consistently after the corresponding result sets. In Section 3.2, we added/confirmed the citation to Table 1 after the summary of study characteristics, including country, sample size, sample type, and periodontal diagnosis.  **Location in revised manuscript.** Results Sections 3.2-3.5, pp. 7-10; Tables 1-4, pp. 11-17.  **Revised text:**  The characteristics of each study… are summarised in Table 1. |
| 3 | Please review the Discussion section and consider expanding it with additional information, especially in sections 4.1, 4.2, and 4.3. | We thank the reviewer for this constructive suggestion. We expanded Sections 4.1–4.3 by adding clearer interpretation of *E. saphenum* within periodontal dysbiosis.  **Location in revised manuscript.** Discussion Sections 4.1-4.3, pp. 17-20.  **Revised text:**  Subgingival biofilm and saliva may capture different aspects of the oral ecosystem. These methodological differences may partly account for the observed patterns of *E. saphenum* detection across studies. |
|  | **Reviewer #8** | **Response** |
| 1 | Dear Editor,  Thank you for your invitation to review the manuscript. The authors have done a good job in the systematic review to evaluate the role of *E. saphenum* in chronic periodontitis despite several challenges. The number of studies which fit into the acceptable criteria are not many. Even among those studies, there is a lot of heterogeneity in sample selection, sample size (especially of healthy volunteers), sample collection, DNA extraction, molecular technique chosen and the method of interpretation. With this background, it is premature at this stage to comment on the role of *E. saphenum* in the etiology of periodontitis. However, one must remember that according to the current concept, the entire dysbiotic microbial community is involved in the initiation, progression and complications of chronic periodontitis with different groups of bacteria playing dominant role in different stages of the disease. Hence, I feel future studies that explore not only the abundance but also the association of *E. saphenum* with other members of the oral microbiome can shed more light on their role in oral health and disease. | We thank the reviewer for this constructive comment. We agree that, given the limited number of eligible studies and the methodological heterogeneity, it is premature to infer an etiologic or causal role for *E. saphenum* in periodontitis. Accordingly, we have revised the manuscript to avoid causal wording and to present *E. saphenum* as a taxon associated with periodontal dysbiosis, particularly in advanced disease, rather than as an independent aetiologic pathogen.  **Location in revised manuscript.** Discussion, pp. 17-22; Limitations and Research Suggestions, p. 22; Conclusion, p. 23.  **Revised text:**  Future studies should also evaluate co-occurrence and network relationships between *E. saphenum* and established periodontal taxa to determine whether it represents a passive marker of anaerobic dysbiosis or a functionally relevant member of pathogenic microbial consortia.  Well-designed longitudinal and interventional studies are required to clarify whether *E. saphenum* contributes functionally to periodontal disease progression or represents an ecological consequence of established disease. |
