# Supplementary material for: Association between Eubacterium saphenum and periodontitis: A systematic review
Source: PLoS One. 2026 Sep 2;21(9):e0357110. doi: 10.1371/journal.pone.0357110 (PMC13537536; doi:10.1371/journal.pone.0357110)
Supplement: Peer review history

# Peer review: Decision Letter 0

**DOI:** 10.1371/journal.pone.0357110.r001

**Authors:** Antonio Magan-Fernandez

2 Jun 2026

Dear Dr. Baharuddin,

Thank you for submitting your manuscript to PLOS ONE. After careful consideration, we feel that it has merit but does not fully meet PLOS ONE’s publication criteria as it currently stands. Therefore, we invite you to submit a revised version of the manuscript that addresses the points raised during the review process.

After careful consideration of the seven reviewer reports, I recommend **major revision** . While the reviewers generally acknowledge the relevance and potential interest of the study, several substantial concerns have been raised regarding the methodology, data analysis, interpretation of results, and clarity of presentation. These issues require significant revision and additional justification before the manuscript can be considered for publication. The authors should address all reviewer comments comprehensively and provide a detailed response explaining the changes made.

Please submit your revised manuscript by Jul 17 2026 11:59PM. If you will need more time than this to complete your revisions, please reply to this message or contact the journal office at plosone@plos.org. When you're ready to submit your revision, log on to https://www.editorialmanager.com/pone/ and select the 'Submissions Needing Revision' folder to locate your manuscript file.

- A letter that responds to each point raised by the academic editor and reviewer(s). You should upload this letter as a separate file labeled 'Response to Reviewers'.
- A marked-up copy of your manuscript that highlights changes made to the original version. You should upload this as a separate file labeled 'Revised Manuscript with Track Changes'.
- An unmarked version of your revised paper without tracked changes. You should upload this as a separate file labeled 'Manuscript'.

If you would like to make changes to your financial disclosure, please include your updated statement in your cover letter. Guidelines for resubmitting your figure files are available below the reviewer comments at the end of this letter.

If applicable, we recommend that you deposit your laboratory protocols in protocols.io to enhance the reproducibility of your results. Protocols.io assigns your protocol its own identifier (DOI) so that it can be cited independently in the future. For instructions see: https://journals.plos.org/plosone/s/submission-guidelines#loc-laboratory-protocols. Additionally, PLOS ONE offers an option for publishing peer-reviewed Lab Protocol articles, which describe protocols hosted on protocols.io. Read more information on sharing protocols at https://plos.org/protocols?utm_medium=editorial-email&utm_source=authorletters&utm_campaign=protocols.

As the corresponding author, your ORCID iD is verified in the submission system and will appear in the published article. PLOS supports the use of ORCID, and we encourage all coauthors to register for an ORCID iD and use it as well. Please encourage your coauthors to verify their ORCID iD within the submission system before final acceptance, as unverified ORCID iDs will not appear in the published article. *Only*  the individual author can complete the verification step; PLOS staff *cannot*  verify ORCID iDs on behalf of authors.

We look forward to receiving your revised manuscript.

Kind regards,

Antonio Magan-Fernandez, DDS, MSc, PhD

Academic Editor

PLOS One

Journal Requirements:

When submitting your revision, we need you to address these additional requirements.

1. Please ensure that your manuscript meets PLOS ONE's style requirements, including those for file naming. The PLOS ONE style templates can be found at https://journals.plos.org/plosone/s/file?id=wjVg/PLOSOne_formatting_sample_main_body.pdf and https://journals.plos.org/plosone/s/file?id=ba62/PLOSOne_formatting_sample_title_authors_affiliations.pdf

2. In the online submission form, you indicated that [The data that support the findings of the study are available from the corresponding author upon reasonable request.].

All PLOS journals now require all data underlying the findings described in their manuscript to be freely available to other researchers, either 1. In a public repository, 2. Within the manuscript itself, or 3. Uploaded as supplementary information.

This policy applies to all data except where public deposition would breach compliance with the protocol approved by your research ethics board. If your data cannot be made publicly available for ethical or legal reasons (e.g., public availability would compromise patient privacy), please explain your reasons on resubmission and your exemption request will be escalated for approval.

3. Please include captions for your Supporting Information files at the end of your manuscript, and update any in-text citations to match accordingly. Please see our Supporting Information guidelines for more information: http://journals.plos.org/plosone/s/supporting-information.

If the reviewer comments include a recommendation to cite specific previously published works, please review and evaluate these publications to determine whether they are relevant and should be cited. There is no requirement to cite these works unless the editor has indicated otherwise.

Additional Editor Comments:

After careful consideration of the seven reviewer reports, I recommend **major revision**. While the reviewers generally acknowledge the relevance and potential interest of the study, several substantial concerns have been raised regarding the methodology, data analysis, interpretation of results, and clarity of presentation. These issues require significant revision and additional justification before the manuscript can be considered for publication. The authors should address all reviewer comments comprehensively and provide a detailed response explaining the changes made.

[Note: HTML markup is below. Please do not edit.]

Reviewers' comments:

Reviewer's Responses to Questions

**Comments to the Author**

1. Is the manuscript technically sound, and do the data support the conclusions?

Reviewer #1: Partly

Reviewer #2: Yes

Reviewer #3: Yes

Reviewer #4: Yes

Reviewer #5: Yes

Reviewer #6: Yes

Reviewer #7: Yes

Reviewer #8: Yes

**********

2. Has the statistical analysis been performed appropriately and rigorously?

Reviewer #1: N/A

Reviewer #2: Yes

Reviewer #3: N/A

Reviewer #4: N/A

Reviewer #5: No

Reviewer #6: Yes

Reviewer #7: Yes

Reviewer #8: Yes

**********

3. Have the authors made all data underlying the findings in their manuscript fully available?

The PLOS Data policy

Reviewer #1: Yes

Reviewer #2: Yes

Reviewer #3: Yes

Reviewer #4: No

Reviewer #5: Yes

Reviewer #6: Yes

Reviewer #7: Yes

Reviewer #8: Yes

**********

4. Is the manuscript presented in an intelligible fashion and written in standard English?

Reviewer #1: Yes

Reviewer #2: Yes

Reviewer #3: Yes

Reviewer #4: Yes

Reviewer #5: Yes

Reviewer #6: Yes

Reviewer #7: Yes

Reviewer #8: Yes

**********

Reviewer #1: Dear authors,

This manuscript presents a systematic review investigating the association between Eubacterium saphenum and periodontitis. The topic is relevant and timely, particularly within the current paradigm of polymicrobial dysbiosis. The manuscript is generally well structured, and the methodology follows recognized standards (PRISMA, PROSPERO registration), which is commendable.

However, several important issues should be addressed to strengthen the scientific rigor and ensure that the conclusions are fully supported by the available evidence.

Major comments

1. The manuscript repeatedly refers to a “consistent association” between E. saphenum and periodontitis. However, as shown in Table 1, the findings across studies are mixed, with several studies reporting no significant association. This weakens the claim of consistency.

The conclusions should be revised to reflect a trend or emerging association, rather than a consistent one.

2. The decision not to perform a meta-analysis is understandable given heterogeneity in sample types, detection methods, and case definitions. However, the justification provided remains limited.

The authors should either strengthen the justification (e.g., explicitly discuss incompatibility of outcome metrics), or provide a semi-quantitative synthesis (e.g., proportion of studies showing positive associations, direction of effects).

3. GRADE assessment likely overestimated

The upgrade to “moderate certainty” appears optimistic given exclusively observational (mostly cross-sectional) data, small sample sizes and methodological heterogeneity.

A more cautious interpretation (e.g., low certainty) should be considered, or the rationale for upgrading should be expanded.

4. While detection methods are described, their limitations are not critically discussed. Important sources of bias include targeted vs untargeted detection approaches, taxonomic resolution issues, pooling vs site-specific sampling and variability in sequencing platforms.

A deeper methodological critique is needed to contextualize the findings.

5. The manuscript appropriately avoids causal claims, but the discussion remains superficial regarding:

reverse causation, ecological shifts vs pathogenic role, whether E. saphenum is a marker or contributor.

The authors should strengthen this section to provide a clearer conceptual positioning.

Minor comments

6. The manuscript is generally understandable but contains several grammatical and typographical errors (e.g., “Then certainty of evidence…”, “dynamicecological”, missing verbs). A thorough language revision by a native English speaker is recommended.

7. The introduction is informative but somewhat broad. Consider sharpening the research gap and more clearly justifying the focus on E. saphenum specifically.

8. Results section is overly descriptive. The addition of simple quantitative summaries (e.g., number of studies supporting the association) would improve clarity.

0. Tables could be more informative

Table 1 is useful but could be enhanced by including effect sizes (if available), statistical significance indicators.

Reviewer #2: 1. Minor correction. Abstract: please check the correct typing of Eubactrium saphenum as its currently written Eubatcriumsaphenum, non-italicized, and without space. Same applies to E. saphenum.

2. Minor correction. In abstract, I suggest clarifying the classification system in the phrase “Studies applying the 2018 classification system consistently reported greater..” by stating 2018 periodontal classification system or referencing the Classification of Periodontal and Peri-Implant Diseases and Conditions.

3. Adding justification. Axis tool was used for quality assessment per section 2.4. Justification needed for not utilizing other assessment tools such as The Joanna Briggs Institute (JBI). I referenced Downes MJ et al. to evaluate different assessment tools, specifically to address Cui et al. 2019, Castillo et al. 2023, Marchensan et al. 2015 cited in the paper.

Ma LL, Wang YY, Yang ZH, Huang D, Weng H, Zeng XT. Methodological quality (risk of bias) assessment tools for primary and secondary medical studies: what are they and which is better? Mil Med Res. 2020 Feb 29;7(1):7. doi: 10.1186/s40779-020-00238-8. PMID: 32111253; PMCID: PMC7049186.

4. Explanation. In the Discussion, Section 4.5 mentions one high quality, Lafaurie et al. 2023, referencing the methodological quality appraisal, which is found in Table 3. Section 3.5 explains Table 2 and Table 3, and in the same section, four papers were given the designation of high quality.

a. If the one study rated as high quality in section 4.5, Lafaurie et al. 2023, is what is meant by strong in association and magnitude as reflected in Odds ratio [12] table 4, please make that it more clear in section 4.5

b. Minor correction: typo Odds ratio footnote table 4

5. In 2.2 Search Strategy, Google Scholar was among the search. Using the author Google Scholar search criteria, I manually searched for khemwong et al 2018. Title: Eubacterium saphenum as a Novel Bacterial Biomarker for Periodontitis Screening, I was not able to find the paper in the first 200 paper or first 20 pages. I used the keyword "Eubacterium saphenum" OR "E. saphenum" OR "E. saphenus" periodontitis OR "periodontal disease". However, Khemwong et al 2018 is available in Google Scholar. Please provide insight into the sensitivity of the search as a potential relevant study may be beneficial to the study and was not cited.

6. In section 3.5, there was an introduction to a new category: high high-quality. Please correct if typo error.

Reviewer #3: Dear Authors,

The manuscript is generally well written, with only a few minor issues related to formatting and style, and the methodological approach appears appropriate. However, the scientific contribution and the amount of knowledge generated from the research question seem somewhat limited. This concern is also reflected in the relatively scarce bibliography supporting the study.

I would suggest incorporating additional bacterial species of major relevance in periodontology, as this could broaden the scope of the analysis, provide greater scientific depth, and strengthen the overall contribution of the manuscript.

Reviewer #4: Introduction

The introduction provides an adequate overview of periodontal dysbiosis and appropriately frames the rationale for investigating non-classical bacterial species in periodontitis. The progression from established periodontal pathogens toward broader dysbiotic models is logically presented. However, some sentences would benefit from grammatical revision and improved clarity. For example, the phrase “Contemporary models emphasise on polymicrobial synergy and dysbiosis” should be revised for grammatical accuracy. Similarly, the sentence describing E. saphenum as “an obligate anaerobes, Gram-positive species” contains grammatical inconsistencies.

The introduction also states that E. saphenum “may serve as biomarkers of disease severity or participate in pathogenic consortia,” which is acceptable as a hypothesis; however, maintaining cautious language throughout the manuscript would improve consistency with the observational nature of the included studies.

Methodology

The methodology section is generally well organized and clearly describes the PECO framework, eligibility criteria, databases searched, and quality assessment methods. However, several clarifications would improve transparency and reproducibility.

The search strategy would benefit from additional detail. Although the databases and search terms are reported, the manuscript does not specify the number of records retrieved from each individual database before deduplication. It would be helpful if the authors included this information either within the PRISMA flowchart or in a supplementary table. Specifically, the number of articles identified from PubMed/MEDLINE, Scopus, Web of Science, Cochrane Library, and Google Scholar should be individually reported. This addition would improve transparency regarding study identification and screening.

The justification for not conducting a meta-analysis is appropriately explained based on heterogeneity in sample type, detection methods, and case definitions. Nevertheless, because the manuscript repeatedly refers to “consistent findings,” it may be useful to clarify that the consistency observed was primarily qualitative across studies using different methodologies.

The section describing quality assessment and certainty of evidence is adequately structured. However, the sentence “four studies of high high-quality” contains a typographical duplication that should be corrected.

Discussion

The discussion appropriately integrates the microbiological findings with current concepts of periodontal dysbiosis and provides a useful comparison between different microbial detection methods. The sections discussing saliva versus subgingival biofilm and the methodological differences between qPCR, HOMIM, sequencing, and checkerboard hybridization are particularly informative.

However, several sentences throughout the discussion would benefit from improved wording and grammatical revision. For example, the expression “dynamicecological changes” contains a typographical error. In addition, there are several formatting inconsistencies involving the presentation of E. saphenum throughout the text.

Some statements in the discussion are more interpretive than directly supported by the included studies. For example, phrases suggesting that E. saphenum may “reinforce” inflammatory microbial communities should be carefully worded to remain consistent with the observational design of the included evidence.

The limitations section is appropriate and acknowledges the predominance of cross-sectional studies, small sample sizes, and methodological heterogeneity. This strengthens the overall balance of the discussion.

Conclusions

The conclusions are generally aligned with the presented findings and appropriately acknowledge that the available evidence does not establish causality. The recommendation for future longitudinal and interventional studies is justified and consistent with the limitations identified in the review.

Grammatical and Typographical Issues

The manuscript would benefit from careful English-language editing to improve readability and grammatical consistency. Examples identified in the current version include:

• “Then certainty of evidence was rated as moderate” should be revised to “The certainty of evidence was rated as moderate.”

• “E. saphenum is an obligate anaerobes” should be revised to “E. saphenum is an obligate anaerobe.”

• “Contemporary models emphasise on polymicrobial synergy” should be revised grammatically.

• “dynamicecological changes” contains a typographical error.

• “high high-quality” contains duplicated wording.

• Minor formatting inconsistencies involving “E.saphenum” versus “E. saphenum” should be standardized throughout the manuscript.

Additionally, the section discussing certainty of evidence and the upgrading of the GRADE assessment would benefit from greater methodological detail regarding the reported odds ratio (OR = 4.85). The manuscript should clarify whether this estimate was obtained from an adjusted multivariable model or from a bivariate analysis only. If the OR was adjusted, the variables included in the adjustment model should be explicitly reported. If it corresponds to an unadjusted association, this limitation should be acknowledged when interpreting the magnitude of effect.

Furthermore, confidence intervals should be consistently reported alongside effect estimates. In particular, the 95% confidence interval (95% CI) corresponding to the reported odds ratio should be included to allow readers to assess the precision of the estimate.

Reviewer #5: Reviewer Report

Manuscript Title: Association between Eubacterium saphenum and Periodontitis: A systematic review

Overall Assessment

This manuscript presents a systematic review evaluating the association between Eubacterium saphenum and periodontitis. The topic is timely and relevant, particularly considering the increasing interest in oral microbial dysbiosis and the role of non-classical periodontal pathogens in disease progression. The manuscript is generally well-structured, follows PRISMA guidelines, and provides a comprehensive overview of currently available observational evidence. The inclusion of AXIS and GRADE assessments strengthens the methodological rigor of the review.

The review highlights a potentially important association between E. saphenum and advanced periodontitis; however, several areas require clarification and refinement before the manuscript can be considered for publication.

Major Comments:

Statistical Interpretation: The manuscript mentions “moderate-to-large effect estimates (OR = 4.85)” but does not sufficiently explain the source study or context of this odds ratio. Please clarify:

• Which study reported this OR.

• Whether the OR was adjusted or unadjusted.

• Confidence intervals and statistical significance.

Minor Comments:

1. Ensure consistency in bacterial nomenclature formatting throughout the manuscript (italicization of species names).

2. Some references require formatting consistency according to journal style.

3. Table formatting can be improved for readability, particularly Tables 2–4.

4. The PRISMA flow diagram should be provided in higher resolution.

5. Consider simplifying lengthy discussion paragraphs for improved readability.

Reviewer #6: Periodontitis is not caused by a single pathogen, but by polymicrobial dysbiosis and the host response. Known pathogens (like P. gingivalis) fail to fully explain disease severity, suggesting that other microbes may be contributing to periodontitis. Eubacterium saphenum has been detected in periodontal pockets and is associated with anaerobic environments. BUT evidence is limited, methods of study are heterogeneous, and it still remains unclear if it is a true contributor of periodontitis or just a more general marker of dysbiosis. Therefore, this study systematically evaluates whether E. saphenum is consistently associated with periodontitis and whether it is correlated with disease severity.

Design: Systematic review (PRISMA 2020 and PROSPERO registered)

Studies included: 8 observational (cross-sectional/case-control)

Sample size: 304 healthy participants and 510 periodontitis participants

Data sources: PubMed, Scopus, Web of Science, Cochrane, Google Scholar

Risk of bias tool: AXIS

Certainty: GRADE

Key Findings:

1. Across 8 studies (814 participants), E. saphenum was consistently more abundant in periodontitis compared to healthy controls, with an odds ratio of 4.85, suggesting a moderate association between E. saphenum and periodontitis

2. This association was directionally consistent across studies despite high heterogeneity, including differences in sampling (biofilm vs saliva) and detection methods (qPCR, 16S sequencing, hybridization platforms)

3. There is evidence of a severity-dependent effect, where E. saphenum abundance increases with disease progression: Stage I to II are associated with lower abundance whereas stage III to IV are associated with higher abundance

4. The overall certainty of evidence was rated as moderate

Strengths

1. Rather than forcing a pooled estimate with high heterogeneity (sampling site, detection method, outcome definitions), the authors avoid generating a potentially misleading summary effect by avoiding meta analysis.

2. The demonstration of a gradient in E. saphenum abundance across disease stages strengthens biological plausibility and provides interesting evidence of a possible dose-response relationship, which is more informative than simple case-control comparisons!

3. The association observed appears to persist across qPCR, 16S sequencing, and hybridization-based methods, suggesting that the trend is not from a single detection technique, which I believe increases the robustness of the findings.

4. The authors seemed to avoid over-attributing causality to E. saphenum and instead discussed it within a broader context of oral dysbiosis, which appears to be consistent with the current understanding of periodontitis and also strengthens the internal validity of their interpretations (and is especially appropriate given the types of studies used for this review).

Major Comments:

1. The biggest limitation is that all the evidence is observational and cross-sectional, so the association is strong but by definition, is not non-causal. You cannot determine whether E. saphenum contributes to disease or is simply a byproduct of dysbiosis.

2. The authors did not appear to fully analyze subgroup or structured comparisons to understand sources of heterogeneity. This heterogeneity may be important to explain variability in findings!

3. There seemed to be some variation in how disease and microbial outcomes are defined, which may have limited comparability across studies

4. Big confounders that can influence the oral microbiome are not really (or at the very least not consistently) controlled for across studies. These may have had large impacts on the oral microbiome.

Minor Comments:

5. The relatively small number of studies limits the strength and generalizability of the conclusions.

6. The narrative approach introduces more subjectivity in the study findings.

Reviewer #7: The manuscript is interesting and addresses a relevant topic. I would suggest a careful English language revision throughout the text. In addition, the tables should be standardized, particularly regarding font style and formatting consistency.

In the Results section, after presenting each set of results, I also recommend citing the corresponding table in parentheses (e.g., Table 1).

Please review the Discussion section and consider expanding it with additional information, especially in sections 4.1, 4.2, and 4.3.

Reviewer #8: Dear Editor,

Thank you for your invitation to review the manuscript. The authors have done a good job in the systematic review to evaluate the role of E. saphenum in chronic periodontitis despite several challenges. The number of studies which fit into the acceptable criteria are not many. Even among those studies, there is a lot of heterogeneity in sample selection, sample size (especially of healthy volunteers), sample collection, DNA extraction, molecular technique chosen and the method of interpretation. With this background, it is premature at this stage to comment on the role of E. saphenum in the etiology of periodontitis. However, one must remember that according to the current concept, the entire dysbiotic microbial community is involved in the initiation, progression and complications of chronic periodontitis with different groups of bacteria playing dominant role in different stages of the disease. Hence I feel future studies that explore not only the abundance but also the association of E. saphenum with other members of the oral microbiome can shed more light on their role in oral health and disease.

**********

what does this mean?). If published, this will include your full peer review and any attached files.

If you choose “no”, your identity will remain anonymous but your review may still be made public.

**Do you want your identity to be public for this peer review?**  For information about this choice, including consent withdrawal, please see our Privacy Policy

Reviewer #1: No

Reviewer #2: **Yes:**  Haider Aljewari

Reviewer #3: No

Reviewer #4: **Yes:**  Irma Yvonne Amaya-Larios

Reviewer #5: No

Reviewer #6: No

Reviewer #7: No

Reviewer #8: **Yes:**  Dr Kishore Bhat

**********

[NOTE: If reviewer comments were submitted as an attachment file, they will be attached to this email and accessible via the submission site. Please log into your account, locate the manuscript record, and check for the action link "View Attachments". If this link does not appear, there are no attachment files.]

To ensure your figures meet our technical requirements, please review our figure guidelines: https://journals.plos.org/plosone/s/figures

You may also use PLOS’s free figure tool, NAAS, to help you prepare publication quality figures: https://journals.plos.org/plosone/s/figures#loc-tools-for-figure-preparation.

NAAS will assess whether your figures meet our technical requirements by comparing each figure against our figure specifications.

# Author comment: Author response to Decision Letter 1

**DOI:** 10.1371/journal.pone.0357110.r002

15 Jul 2026

We made the revision as requested. Thanks

Attachment Submitted filename: Response to Reviewers.docx

# Peer review: Decision Letter 1

**DOI:** 10.1371/journal.pone.0357110.r003

**Authors:** Antonio Magan-Fernandez

13 Aug 2026

Association between Eubacterium saphenum and Periodontitis:

A systematic review

PONE-D-26-18732R1

Dear Dr. Baharuddin,

We’re pleased to inform you that your manuscript has been judged scientifically suitable for publication and will be formally accepted for publication once it meets all outstanding technical requirements.

Within one week, you’ll receive an e-mail detailing the required amendments. When these have been addressed, you’ll receive a formal acceptance letter and your manuscript will be scheduled for publication.

An invoice will be generated when your article is formally accepted. Please note, if your institution has a publishing partnership with PLOS and your article meets the relevant criteria, all or part of your publication costs will be covered. Please make sure your user information is up-to-date by logging into Editorial Manager at Editorial Manager® and clicking the ‘Update My Information' link at the top of the page. For questions related to billing, please contact billing support.

If your institution or institutions have a press office, please notify them about your upcoming paper to help maximize its impact. If they’ll be preparing press materials, please inform our press team as soon as possible -- no later than 48 hours after receiving the formal acceptance. Your manuscript will remain under strict press embargo until 2 pm Eastern Time on the date of publication. For more information, please contact onepress@plos.org.

Kind regards,

Antonio Magan-Fernandez, DDS, MSc, PhD

Academic Editor

PLOS One

Additional Editor Comments (optional):

Authors have addressed the comments made by the reviewers and the manuscript is now acceptable for publication.

Reviewers' comments:

Reviewer's Responses to Questions

**Comments to the Author**

Reviewer #1: All comments have been addressed

Reviewer #3: All comments have been addressed

**********

2. Is the manuscript technically sound, and do the data support the conclusions?

Reviewer #1: Yes

Reviewer #3: Yes

**********

3. Has the statistical analysis been performed appropriately and rigorously?

Reviewer #1: Yes

Reviewer #3: N/A

**********

4. Have the authors made all data underlying the findings in their manuscript fully available?

The PLOS Data policy

Reviewer #1: Yes

Reviewer #3: Yes

**********

5. Is the manuscript presented in an intelligible fashion and written in standard English?

Reviewer #1: Yes

Reviewer #3: Yes

**********

Reviewer #1: Dear authors,

Thank you for considering my comments in your revised version tha tis now publishable

Best regards

Reviewer #3: Dear authors:

The revised version of the manuscript demonstrates a substantial improvement in its overall scientific quality, rigor, and clarity. The authors have addressed the reviewers' comments comprehensively and thoughtfully, resulting in a significantly strengthened manuscript.

The revisions have considerably enhanced the scientific value of the study. The Results section is now presented in a clearer and more structured manner, allowing readers to better understand the main findings and appreciate their relevance. Likewise, the Discussion has been notably strengthened through a more critical interpretation of the findings, a better integration of the results with the existing body of evidence, and a more balanced consideration of the clinical and scientific implications of the study.

Particularly commendable is the improvement in the Limitations section, which now provides a more transparent and realistic appraisal of the study's constraints. This addition increases the credibility of the work by demonstrating the authors' awareness of the methodological boundaries while appropriately contextualizing the interpretation of the findings.

Overall, the manuscript has evolved into a scientifically robust and well-balanced contribution. The revisions have substantially increased its methodological transparency, strengthened the interpretation of the findings, and enhanced its potential impact on the field of dental research. The final version represents a valuable contribution to the scientific literature and provides meaningful evidence that will be of interest to both researchers and clinicians.

Best regards,

**********

what does this mean?). If published, this will include your full peer review and any attached files.

If you choose “no”, your identity will remain anonymous but your review may still be made public.

**Do you want your identity to be public for this peer review?**  For information about this choice, including consent withdrawal, please see our Privacy Policy

Reviewer #1: No

Reviewer #3: No

**********

# Editor report: Acceptance letter

**DOI:** 10.1371/journal.pone.0357110.r004

**Authors:** Antonio Magan-Fernandez

PONE-D-26-18732R1

PLOS One

Dear Dr. Baharuddin,

I'm pleased to inform you that your manuscript has been deemed suitable for publication in PLOS One. Congratulations! Your manuscript is now being handed over to our production team.

At this stage, our production department will prepare your paper for publication. This includes ensuring the following:

* All references, tables, and figures are properly cited

* All relevant supporting information is included in the manuscript submission,

* There are no issues that prevent the paper from being properly typeset

You will receive further instructions from the production team, including instructions on how to review your proof when it is ready. Please keep in mind that we are working through a large volume of accepted articles, so please give us a few days to review your paper and let you know the next and final steps.

Lastly, if your institution or institutions have a press office, please let them know about your upcoming paper now to help maximize its impact. If they'll be preparing press materials, please inform our press team within the next 48 hours. Your manuscript will remain under strict press embargo until 2 pm Eastern Time on the date of publication. For more information, please contact onepress@plos.org.

You will receive an invoice from PLOS for your publication fee after your manuscript has reached the completed accept phase. If you receive an email requesting payment before acceptance or for any other service, this may be a phishing scheme. Learn how to identify phishing emails and protect your accounts at https://explore.plos.org/phishing.

If we can help with anything else, please email us at customercare@plos.org.

Thank you for submitting your work to PLOS One and supporting open access.

Kind regards,

PLOS One Editorial Office Staff

on behalf of

Prof. Antonio Magan-Fernandez

Academic Editor

PLOS One
